# Species Distribution Models for Crop Pollination: A Modelling Framework Applied to Great Britain

**DOI:** 10.1371/journal.pone.0076308

**Published:** 2013-10-14

**Authors:** Chiara Polce, Mette Termansen, Jesus Aguirre-Gutiérrez, Nigel D. Boatman, Giles E. Budge, Andrew Crowe, Michael P. Garratt, Stéphane Pietravalle, Simon G. Potts, Jorge A. Ramirez, Kate E. Somerwill, Jacobus C. Biesmeijer

**Affiliations:** 1 School of Biology, University of Leeds, Leeds, United Kingdom; 2 Department of Environmental Science, Aarhus University, Roskilde, Denmark; 3 Naturalis Biodiversity Center, Leiden, Netherlands; 4 Institute for Biodiversity and Ecosystem Dynamics, University of Amsterdam, Amsterdam, Netherlands; 5 Food and Environment Research Agency, Sand Hutton, York, United Kingdom; 6 School of Agriculture, Policy and Development, Reading University, Reading, United Kingdom; 7 School of Geography, University of Leeds, Leeds, United Kingdom; CNR, Italy

## Abstract

Insect pollination benefits over three quarters of the world's major crops. There is growing concern that observed declines in pollinators may impact on production and revenues from animal pollinated crops. Knowing the distribution of pollinators is therefore crucial for estimating their availability to pollinate crops; however, in general, we have an incomplete knowledge of where these pollinators occur. We propose a method to predict geographical patterns of pollination service to crops, novel in two elements: the use of pollinator records rather than expert knowledge to predict pollinator occurrence, and the inclusion of the managed pollinator supply. We integrated a maximum entropy species distribution model (SDM) with an existing pollination service model (PSM) to derive the availability of pollinators for crop pollination. We used nation-wide records of wild and managed pollinators (honey bees) as well as agricultural data from Great Britain. We first calibrated the SDM on a representative sample of bee and hoverfly crop pollinator species, evaluating the effects of different settings on model performance and on its capacity to identify the most important predictors. The importance of the different predictors was better resolved by SDM derived from simpler functions, with consistent results for bees and hoverflies. We then used the species distributions from the calibrated model to predict pollination service of wild and managed pollinators, using field beans as a test case. The PSM allowed us to spatially characterize the contribution of wild and managed pollinators and also identify areas potentially vulnerable to low pollination service provision, which can help direct local scale interventions. This approach can be extended to investigate geographical mismatches between crop pollination demand and the availability of pollinators, resulting from environmental change or policy scenarios.

## Introduction

The importance of ecosystems to human well-being was documented by the Millennium Ecosystem Assessment, which also recognised that the majority of pollinators are in decline or threatened [1]. Crop pollination is a key ecosystem service vital to the maintenance of both wild plant communities and agricultural productivity. Over three quarters of the world's major crops benefit from insect pollination, with an economic value estimated to be around € 153 billion globally in 2005 and approximately € 500 million in the United Kingdom [2]–[4]. Pollination services are mainly provided by wild pollinators (bees, hoverflies, flies, moths, beetles) and domesticated bees (primarily honey bee *Apis mellifera*). The recent declines observed in pollinators, mainly bees [5], [6], may therefore impact on the production of and profits from pollinator-dependent crops. For instance, long-term trends of global crop production suggest that to compensate for a 3–8% yield reduction expected in absence of animal pollination, the expansion of agricultural land would be much greater (ca. 25%, and proportionally much greater in the developing world), which in turn could accelerate habitat destruction and contribute to further pollination loss [7].

Knowing spatial patterns of managed and wild pollinators is therefore crucial to estimate their availability to crops and to inform management strategies. In general, however, we have incomplete knowledge of where wild pollinators occur. To overcome this, a recent approach proposed by Lonsdorf *et al.* [8] derives the probability of occurrence of wild bees from a relative availability (from 0 to 1) of nesting sites and floral resources within a landscape, assessed for a few large guilds of species. This probability is then used to derive the relative pollinator service available to a particular crop, taking into account crop location, its potential pollinators and their foraging distance.

Here we propose an approach that combines the Lonsdorf model to derive pollinator services, with predicted pollinator occurrences from species records rather than from landscape suitability. One of the preferred tools to predict species spatial patterns from geographically and temporally sparse biodiversity data are species distribution models (SDMs), which now offer a wide range of approaches due to enhanced computational resources, increasing availability of spatially explicit environmental information and accessibility of species occurrence databases [9], [10]. SDMs mainly differ in the requirements of the species records (e.g. presence and true absence, presence and background, presence only) and in the algorithms used to define the species niche as a function of the predictors (e.g. regression methods, machine learning techniques, Bayesian statistics) [11]. While it is unlikely that a single modelling approach will outperform all others in any situation, comparative work [10], [12] helps to identify the main elements affecting model performance, and thus represents a valuable resource to orient the end user in the choice of the modelling approach. In this study we use the maximum entropy method implemented within the freely available software MaxEnt [13], to derive species distributions from sparse pollinator records. MaxEnt is a general purpose machine-learning technique that estimates the potential distribution of the species by estimating the probability distribution of maximum entropy (i.e. that is most spread out), subject to the constraints derived from the available occurrence data [13]. MaxEnt has received increasing attention within the field of SDM (File S1: Fig. S1–1), both in single species applications [14], [15] and in comparisons of algorithms [16], [17]. Extensive experimental work has allowed guidance for several settings of MaxEnt modelling [18], as well as drawing attention to the main elements affecting its performance [19]–[21].

First, we describe the main steps and tests carried out to calibrate the MaxEnt model; we then show the predictions of the calibrated model for a representative sample of crop-pollinators within Great Britain; finally, we use the predicted species distributions to derive the potential pollination service of wild pollinators and managed honey bees, using the annual legume field bean *Vicia faba* as a test case. In conclusion, we discuss some of the methodological advantages of our approach, the remaining challenges and how it can be further applied to other ecological questions.

## Materials and Methods

### Datasets

#### Wild pollinator data

We used presence-only records of wild bees and hoverflies collected within the period 2000–2010 (“Bees, Wasps and Ants Recording Society”, BWARS [22]; “Hoverfly Recording Scheme”, HRS [23]). The spatial accuracy of the data varied between 10 m and 10 km; we chose 1 km^2^ as a suitable resolution to balance the aim to derive patterns at a national extent as well as to inform decisions at the local scale. We registered all records with accuracy finer than or equal to 1 km to a grid of 1 km^2^ cells, removing duplicates so that within each cell there was only one record of the same species. We use the term “*records*” to mean the number of original records for each species, with accuracy finer or equal to 1 km, whilst we use “*occurrences*” to denote the number of 1 km^2^ grid cells occupied by a species.

To calibrate the model, we selected a subset of pollinator species representing a range of geographic distributions. To follow a repeatable and objective procedure, we used hierarchical cluster analysis to group the species based on the number of occurrences, minimum and maximum latitude and longitude (expressed as northing and easting on the British National Grid), and spatial distribution. Spatial distribution was measured as the longest distance within the third quartile of the pairwise distances between the occurrences of each species; we preferred the third quartile over the fourth quartile, to avoid potential outliers and thus obtain a better characterisation of the species' distribution extent [24]. We selected about one third of the species from within each resulting cluster, ensuring representation across genera. Finally, we used visual inspection of the species occurrence maps to confirm they represented contrasting geographic distributions (e.g. ranging from few to many occurrences, and from narrow to wide range). The selection was carried out separately for bees and hoverflies.

Six hoverfly species and 22 bee species were selected (File S2: Table S2–1). The number of occurrences ranged from 232 to 4048 for hoverflies and from 12 to 4144 for bees, and longest third quartile distance ranged from 215.4 km to 312.6 km for hoverflies and from 63.8 km to 763.9 km for bees.

#### Managed pollinator data

The distribution of the managed honey bees was derived from the optional beekeeping register BeeBase, held by the National Bee Unit at the Food and Environment Research Agency [25]. The number of bee foragers was modelled at the 4 km^2^ resolution from the size and location of apiaries in England and Wales using the estimated average foraging distance [26]–[28]. At the time of this study, a sufficient coverage of apiaries from which to model forager numbers was not available for Scotland. To match the grain to the other pollinator data, the number of foragers was divided by four, assuming a uniform distribution across the four 1 km^2^ grid cells. The data were then linearly rescaled between 0 and 1, to provide a relative score of honey bee foragers per km^2^.

#### Environmental predictors

We use four types of environmental predictors (Table 1):

**Table 1:** Environmental predictors used to derive species distribution models.

Variable theme	Variable name	Variable definition
Topography	*AspNS	Aspect = sin (rad (aspect))
	†AspEW	Aspect = cos (rad (aspect))
Climate	‡Isoth	Isothermality %
	TAR	Temperature Annual Range
	MTDQ	Mean Temperature of Driest Quarter
	MTCQ	Mean Temperature of Coldest Quarter
	RainSeasCV	Precipitation Seasonality (Coefficient of Variation)
	RainCQ	Precipitation Coldest Quarter (mm)
Land-cover	BLW	Broadleaf woodland
	ConW	Coniferous woodland
	AR	Arable
	GrassImp	Improved grassland
	GrassSN	Semi-natural grassland
	MHB	Mountain, heath, bog
	SW	Saltwater
	FW	Freshwater
	Coast	Coastal
	UrbGar	Built-up areas and gardens
Pesticides	Pest	Average number of risk hectares

* AspNS  =  sine (radiant [aspect angle in degree]); †AspEW  =  cosine (radiant [aspect angle in degree]); ‡Isothermality %  =  Mean Diurnal Range (MDR)/Temperature Annual Range (TAR); where MDR  =  Mean of monthly (max temp – min temp)); TAR  =  Max Temperature of Warmest Month – Min Temperature of Coldest Month. Isothermality is a quantification of how large the day-to-night temperature oscillation is in comparison to the summer-to-winter oscillation. A value of 100 would represent a site where the diurnal temperature range is equal to the annual temperature range. A value of 50 would indicate a location where the diurnal temperature range is half of the annual temperature range.

- Land cover classes: 10 continuous variables representing cover percent for Great Britain, from CEH Land Cover Map 2007 [29].
- Bio-climatic data [30]: six variables derived from 25 km^2^ gridded monthly averages of minimum temperature, maximum temperature and precipitation for the 1991–2000 period (to provide an average climate characterizing the decade preceding the oldest species records), obtained from UKCP09 [31] and resampled to 1 km^2^ grain. Data were computed within R software environment 2.13.0 [32].
- Topography: two indices describing aspect (i.e. slope orientation), derived from a 10 m horizontal interval digital elevation model [33] resampled to 1 km^2^.
- Pesticides: treated hectares that pose a potential risk to bees per hectare of crop grown, derived from the Pesticides Usage Survey [34] and linked to cropping data from the Defra June Agricultural Survey [35]. The impact was assessed for honey bees, due to data availability for this species [36], [37].

Bio-climatic and topographic variables were selected using Jolliffe's Principal Component Analysis [38] to minimize multicollinearity [39]. Details of this procedure and the Pearson's correlation between the chosen variables are in File S2: Table S2–2.

### Species distribution models

#### Choice of the background data

Species distribution models were carried out within MaxEnt 3.3.3 k [13], [40]. One of the advantages of MaxEnt is that presence-only data can be used. In this case the MaxEnt probability is defined over a sample of points taken from the study region (“background points”) which may, or may not, contain the species presence records [13].

To derive the MaxEnt probability, in addition to the environmental conditions at localities where a species is found, the model requires a sample from the background. This assumes a uniform survey effort over the entire study area, but if this assumption is violated the background information should reflect the sample bias. A possible correction is to restrict the selection of the background points to a region where a target group of species has been observed by similar methods [19]. We tested for violation of this assumption by comparing the AUC (Area Under the Curve of the Receiver Operating Characteristic) of models based on all known records of crop pollinators in Great Britain (i.e. the target group background, TGB), against the AUC of models based on *n*-time sampling an equal number of points from the entire study area (referred as null models) [41]. We found that the average AUC of 10 sets of 5000 points drawn from the TGB was significantly greater than the average AUC of 100 null models from the entire study area (0.771±0.002 and 0.543±0.005 respectively). The background localities for the individual SDMs were therefore drawn from within the TGB.

#### Model calibration

During the model calibration we evaluated the single and combined effects of changing two main MaxEnt settings:

- Choice of the feature classes (the functions) used to fit the data: default settings currently allow for six feature classes (*Linear*, *Quadratic*, *Product*, *Threshold*, *Hinge* and *Categorical*), provided sufficient samples are available [42]. For each species, we compared models with default settings against models built with *Hinge* features alone, which are base functions for piecewise linear splines [18]. When using *Hinge* alone, we modified the threshold for minimum sample size to 12 (rather than 15, the default) to allow its application to the full set of calibration species (which included *Andrena niveata* with 12 records and *Lasioglossum semilucens* with 13).
- Default prevalence: prevalence is defined as the probability of presence at ordinary occurrence points and, when absence data are not available, MaxEnt assigns it 0.5 [18]. It is defined over specific spatial and temporal scales, which should be taken into account particularly when working with pools of species differing in their rarity [42]. For each species, we compared models with default prevalence 0.5 to models where this value was modified to reflect the species commonality relative to the rest of the species within the pollinator set. To our knowledge there are no theoretically based rules to adjust this value; we therefore empirically rescaled it, considering the number of available records, the number of occurrences and the number of years with non-zero observations over the temporal scale (2000–2010). Each species was then assigned a new prevalence, from 0.1 to 0.5 (File S3: Table S3–1, Fig. S3–1, Figs S3–2.1 and S3–2.2).

#### Model performance

We evaluated the effects of changing default settings on model calibration with two metrics:

- The model testing AUC and its standard deviation (AUC_SD_), using mixed effect models with species as random factor. We tested whether changing default parameters significantly affected the AUC and its variability between different models.
- The standard deviation (SD) of the *Permutation importance (%)* between predictors and background, using generalized linear models. The *Permutation importance* is derived by randomly permuting the values of each predictor between presence and background in turn; the model is re-evaluated on the permuted values and the resulting drop in training AUC is then normalized to percentages. We expected that a model with good discriminatory power would result in a greater spread between the significance of the different predictors.

All models were carried out through *k*-fold cross-validation, where data are divided into *k* mutually exclusive subsets: for each run, *k*–1 of them are combined into a set for training, and one is used for the prediction (i.e. model testing). The number of mutually exclusive subsets was 10 for all species. Evaluation was performed on the average of the cross-validation runs (for AUC, AUC_SD_, Permutation importance and its SD).

After completing the model calibration, we used null models to test whether the resulting SDMs provided a significantly better fit than expected by chance alone. With presence-only data the maximum achievable AUC is <1 [43]: namely, it is 1-*a*/2, with *a* being the true fraction of the study area occupied by a species, typically unknown when absence data are not available [13]. To assess SDM accuracy, therefore, we compared the average AUC value of each species SDM (AUC_SDM_) with the average AUC value of a set of null models (AUC_NM_) where species records were replaced by randomly chosen locations [41]. We expected AUC_SDM_ > AUC_NM_.

Following the assessment of model performance, we tested whether the predictors that were most important for fitting the training data were also the most important for predicting species distribution. Single-predictor models are built within MaxEnt for the training and testing phases: we ranked them according to their gain (a measure of model fit), assigning one the model with the lowest increase in gain. We then computed the Spearman's rank correlation between training and testing models for each predictor, using *Mean* and *Mode*. Their observed correlations were tested against the frequency of randomly generated correlations, using 999 bootstrap replicates [44]. Lastly, we also tested whether the *Mean* of each predictor was correlated to its *Mode*, for the pooled set of training and testing models.

### Application to crop pollinators

#### Pollinator distribution models

The settings chosen from the model calibration were used to derive SDMs for the wild pollinators of field bean. We used expert knowledge from our team and existing literature [45] to select species known to pollinate field bean. For each species, we used “*10^th^ percentile training presence*” as threshold to derive a binary map (1 = presence, 0 =  absence) from the predicted continuous probability of each of the cross-validation runs. We summed together the 10 binary maps and we took the areas where the sum equalled 10 (i.e. areas where all 10 runs had predicted presence) as the presence area for that species. This strict criterion implies that the sites where all 10 runs have predicted presence identify conditions of greatest suitability for the species. The effects of this choice compared to a less conservative criterion are presented in the results. We then assigned to each presence area the average probability of presence derived from the 10 model runs, this became the predicted likelihood of occurrence for that particular species. This map was used as pollinator source to derive the potential pollinator service.

For consistency with the modelled distributions of wild pollinators, we applied a threshold to the probability of occurrence of managed honey bees to distinguish absence from presence. We used the fifth percentile as a cut-off, corresponding to a 0.001 probability of occurrence, and we assigned “absence” to areas with probability below this threshold. This threshold is less conservative than the one used for wild pollinators, to reflect the fact that the data on managed pollinators are based on information updated annually and on dispersal functions empirically derived.

#### Crop distribution

We used distributional records of field bean from the Defra 2010 June Agricultural Survey and mapped to an original grain of 4 km^2^, which we resampled to 1 km^2^ to match the grain of the SDMs.

#### Pollinator service

We adapted the model by Lonsdorf *et al.* [8], which focuses on wild bees. The model maps an index of potential pollinator abundance (“pollinator source map”), based on the relative availability of nesting sites and floral resources across the landscape as provided by expert knowledge and/or field observations. The source map is used to estimate the potential pollinator service ***P_os_*** [8]:
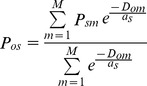
(1)Where: ***P_sm_***  =  relative index for pollinator species ***s*** on map unit ***m***, based on the pollinator source map; ***D_om_***  =  (Euclidean) distance between map unit ***m*** and crop cell ***o***; ***a_s_***  =  average foraging distance of species ***s***. Equation 1 is the distance-weighted proportion of ***M*** cells occupied by foraging pollinators [46]. The score ***P_os_*** therefore represents the relative abundance (from 0 to 1) of the pollinator species ***s*** visiting each crop cell, i.e. the pollination service from species ***s***.

The main difference between Lonsdorf's model and ours is the input used to generate the potential pollinator source (*P_sm_*): in our case, it is not derived from landscape suitability scores for nesting sites and floral resources, but from SDMs based on actual species records. We discuss the implications later in the text.

The total service ***P_o_*** of ***S*** pollinator species visiting cell ***o*** is [8]:
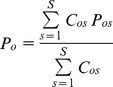
(2)Where ***C_os_*** is 1 if the crop on farm ***o*** requires pollinator ***s***, and 0 otherwise.

The model was carried out in NetLogo 5.0.1 [47]; the outputs were exported to ArcGIS 10.0 [48] for visualization.

Wild pollinator foraging distances were estimated from expert knowledge within our team and existing literature [49], [50]: we used 1 km for *Andrena labialis, A. wilkella, Bombus hortorum, B. lucorum, B. muscorum and Osmia rufa*; we doubled this distance for*B. lapidarius*, *B. pascuorum* and *B. terrestris*. We used the estimated foragers' occurrence on the crop parcels as a proxy for the service provision by managed pollinators, as this dataset already accounted for their typical foraging distance.

## Results

### Model calibration

Modifying default settings for feature class and prevalence did not significantly affect model performance (AUC) (P>0.5 for all, File S4: Table S4–1); variability between cross-validation runs (AUC_SD_) was also not affected, with the exception of modifying prevalence for features class *All* in hoverflies, which increased AUC_SD_ (File S4: Table S4–2). In contrast, the ability to discriminate the importance of the different predictors, measured by the SD of the *Permutation importance (%)* was greater in models built using *Hinge* feature class alone (P≤0.001 in bees and hoverflies, File S4: Table S4–3); within bees this effect was even stronger when *Hinge* was used in combination with modified prevalence. In addition, the more complex response curves allowed by the default settings *All* suggested in some cases a possible overfit (a representative subset of these curves is shown in File S4: from Fig. S4–1.1 to Fig. S4–1.4).

Based on these patterns we chose *Hinge* feature class alone (with modified prevalence) to derive SDMs for the set of pollinators relevant to British crops.

### Model performance

**Figure 1:**
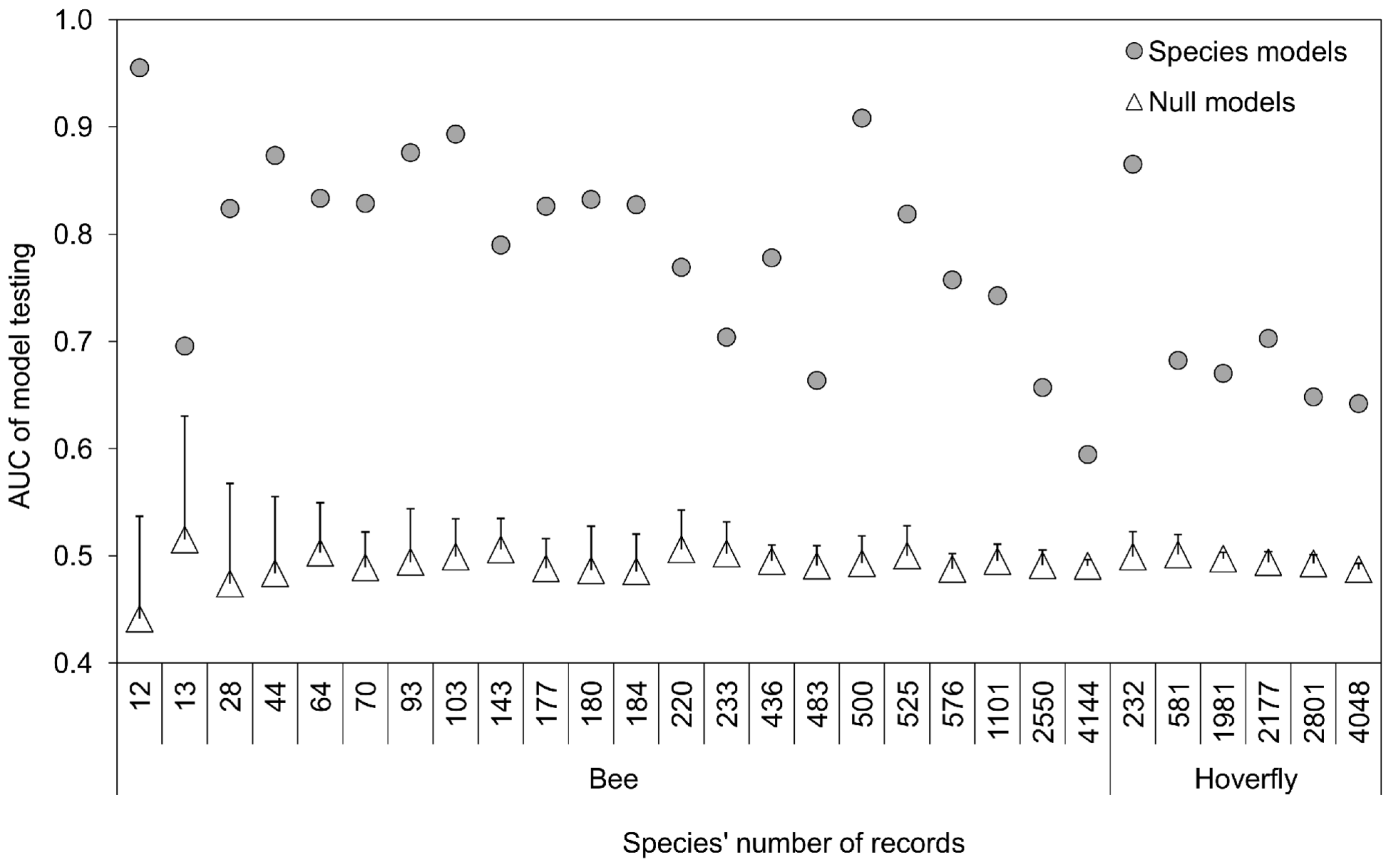
Performance of the calibrated SDMs against performance of the null models. Model performance is measured as the AUC of model testing. Error bars show the SD of the null models (10 sets for each species, each modelled with 10-fold cross-validation). The number of available records is used to plot different species along the *x*-axis.

SDMs provided a significantly better fit than expected by chance alone for all the species (Fig. 1 shows the results for the AUC of the testing phase; a similar pattern was observed for the AUC of the training phase).

**Figure 2:**
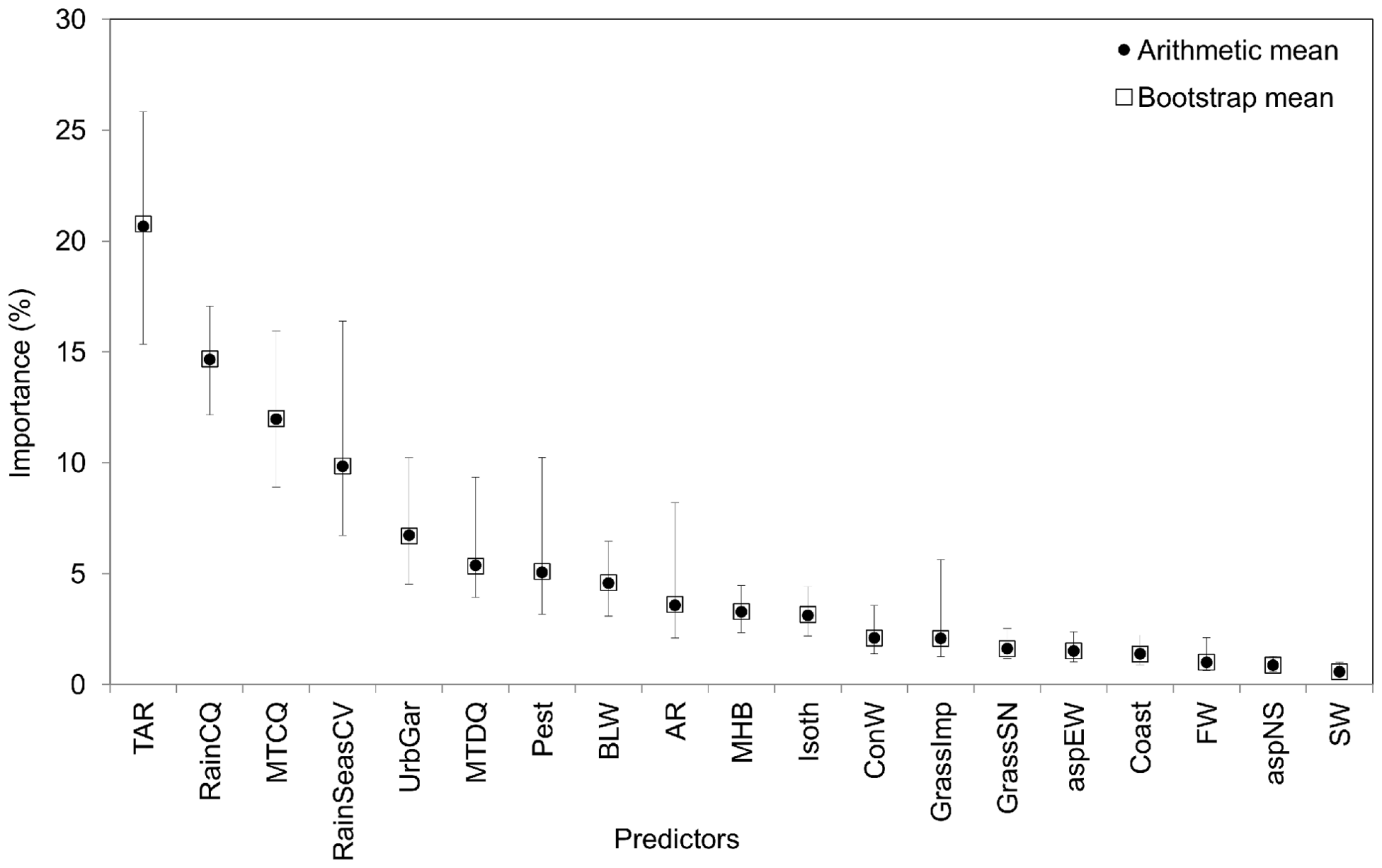
Importance of different predictors. Arithmetic and bootstrap mean and 95% confidence interval of each predictor's importance, pooled across species. Confidence interval shows the 95% biased-corrected accelerated percentile, based on 999 replicates. Predictors are defined in Table 1.

Of the predictors tested (Table 1), climatic variables generally ranked higher than the others, although with variation between species. In particular, *Temperature Annual Range* (TAR), *Precipitation of the Coldest Quarter* (RainCQ), *Mean Temperature of the Coldest Quarter* (MTCQ) and *Precipitation Seasonality* (RainSeasCV) were the predictors with the greatest importance (Fig. 2 and File S5: from Fig. S5–1.1 to Fig. S5–1.4).

The *Mean* and *Mode* of the predictors' importance were significantly correlated between training and testing phase (ρ_Mean_  = 0.974; ρ_Mode_  = 0.944; File S5: Figs S5–2 and S5–3). The correlation between *Mean* and *Mode* of the pooled set of training and test models across species was also significant (ρ = 0.940; File S5: Figs S5–3 and S5–4).

### Pollinator distribution models

**Figure 3:**
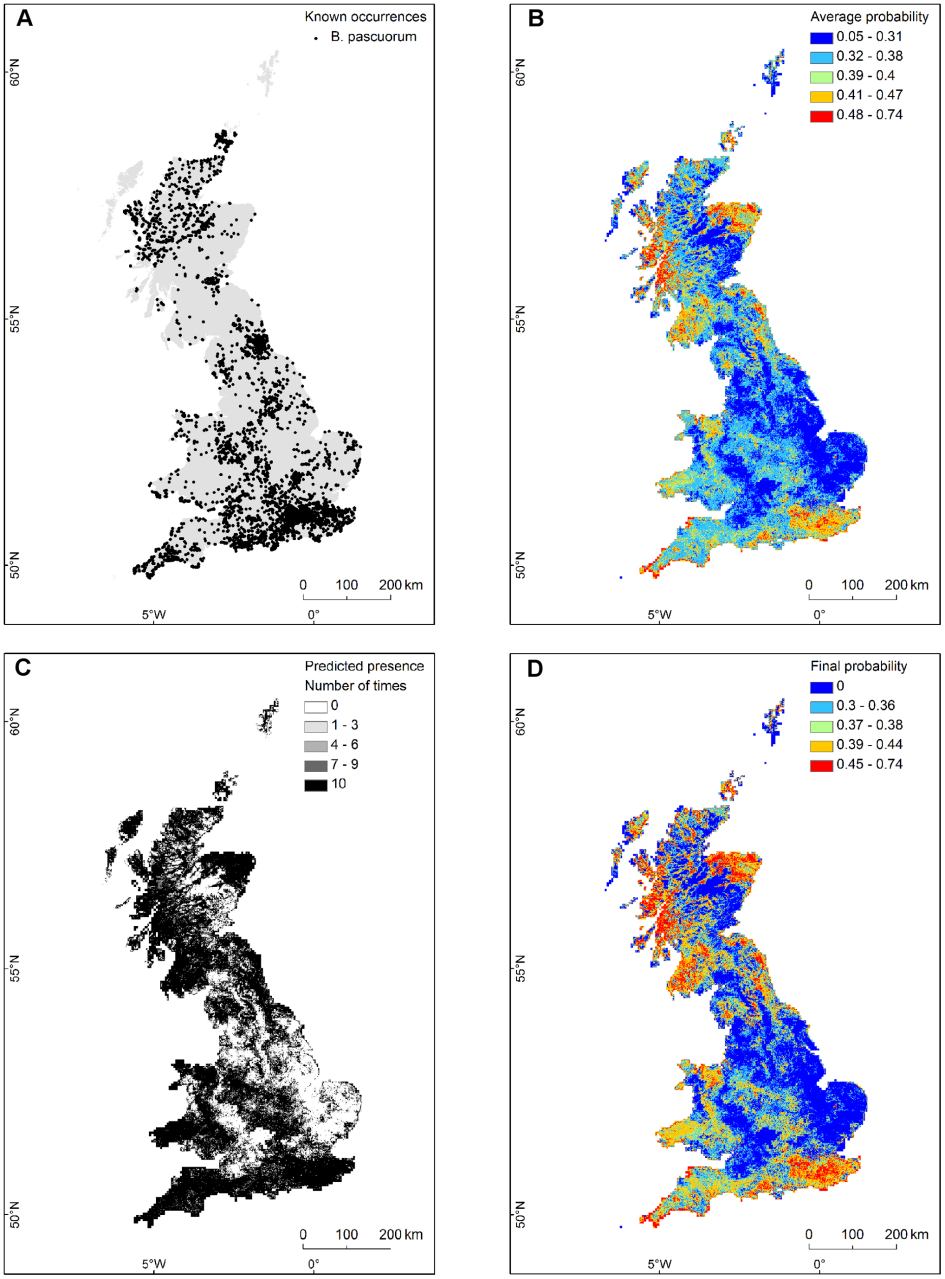
SDM outputs for *Bombus pascuorum*. Outputs from the SDM for *B. pascuorum*: (A): known occurrences; (B): predicted MaxEnt average probability from the 10-fold cross-validation models, using geometric interval classes from blue to red; (C): summed presence from the 10 binary maps (10 indicates areas where all 10 models predicted presence and 0 areas where all models predicted absence); (D): final predicted probability for *B. pascuorum* used as input for the pollinator service, derived from assigning the average probability values in (C) only to the areas where all models predicted presence, and 0 to any area predicted “absence” by at least one binary map. Map projection: British National Grid (BNG).

Figure 3 shows an example of outputs for *Bombus pascuorum*, one of the pollinator species of field beans. The average probability of presence from the 10 cross-validation models ranged from 0.05 to 0.74. The fraction of the 4144 occurrences available for *B. pascuorum* predicted as presence after converting each model prediction into a binary map (using 10^th^ percentile training presence as threshold) was 0.90±0.003 (mean ± SD). This fraction decreased to 0.86 when 0 was assigned to any area predicted absence by at least one binary map, while retaining the average probability only in areas identified as “presence” by all 10 binary maps.

Across species, the average fraction of observed occurrences captured within each species' final area of presence was 0.84±0.030 (mean ± SD). This fraction was positively but non-significantly correlated with the number of available occurrences (Spearman ρ = 0.64, significance assessed with 1000 permutations of samples without replacement, yielding a frequency ∼0.06). Across species, the average final area of presence was 16%±9% smaller than the average from the 10 runs, and negatively correlated with the number of species occurrences (Spearman ρ = −0.85, observed with a frequency ∼0.005 from 1000 permutations). Had we derived the final area of presence from sites predicted by at least nine runs rather than by all 10 runs, the fraction of captured occurrences would be on average 3% greater (± 2%) than the one obtained with the stricter criterion, and negatively correlated with the number of species occurrences (Spearman ρ = −0.86, observed with a frequency ∼0.005 from 1000 permutations).

### Pollinator service

**Figure 4:**
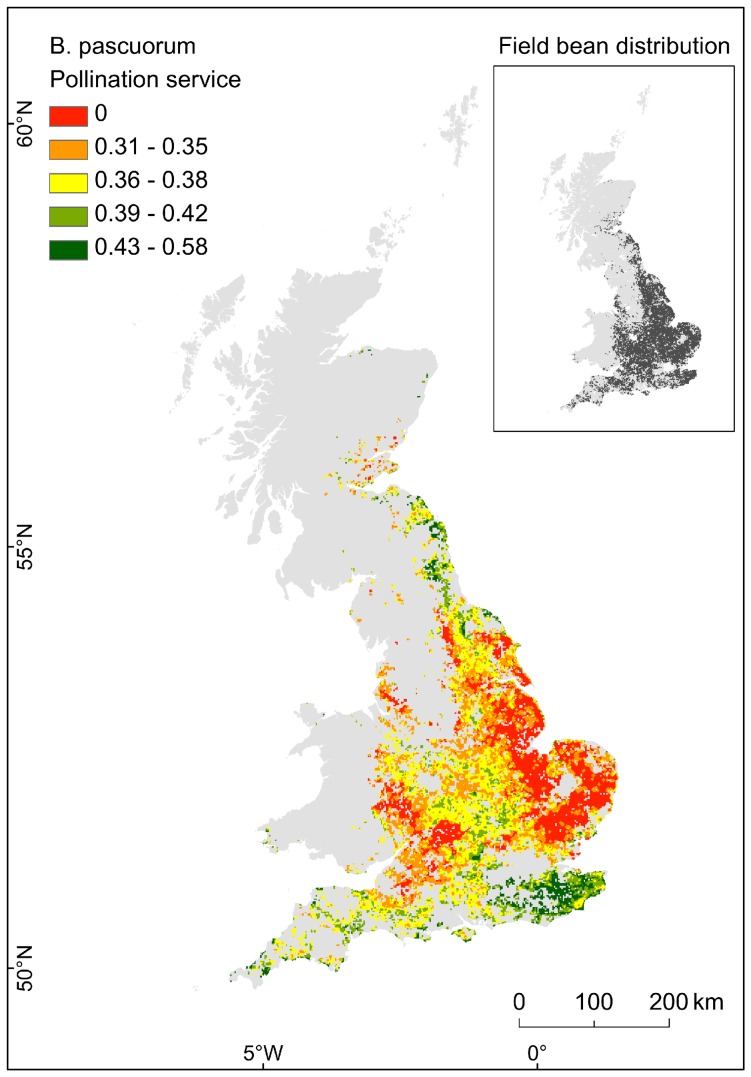
Pollination service to field beans, from *Bombus pascuorum*. The potential pollination service is represented using geometric intervals, with the exclusion of the zero class which was manually defined. Areas evaluated as 0 indicate crop fields outside the foraging distance of *B. pascuorum* (i.e. no pollination service). Map projection: BNG.

Figure 4 shows an example of potential pollinator service to field bean for *Bombus pascuorum,* as relative scores from 0 to 1. Predictions ranged from 0 to 0.58 and areas evaluated as zeroes indicate crop fields outside the typical foraging distance of *B. pascuorum* (i.e. no pollination service). Results for the remaining wild pollinators of field bean are in File 6: from Fig. S6–1 to Fig. S6–8.

**Figure 5:**
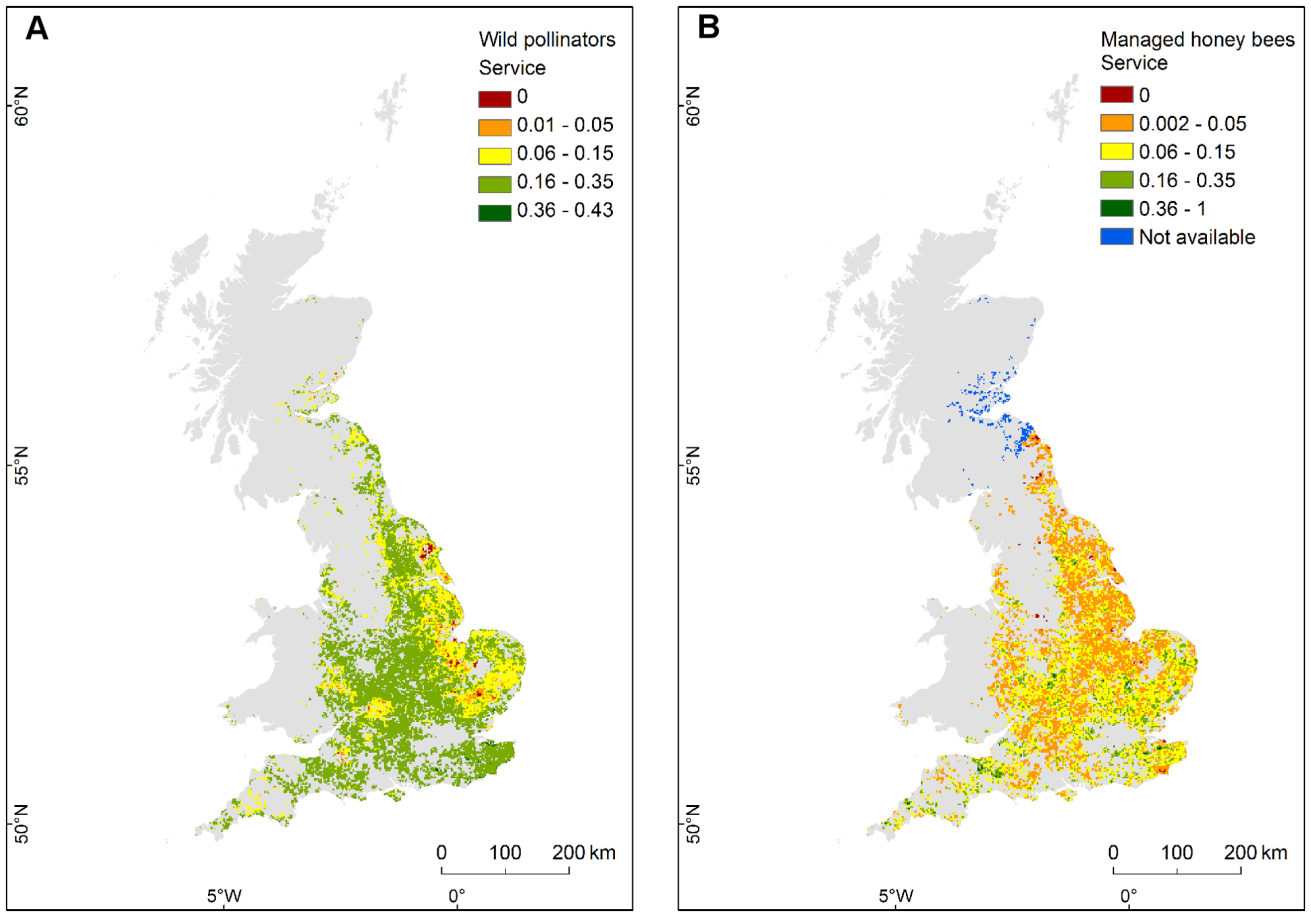
Pollination service to field beans, from wild and managed pollinators. Maps show the potential pollination service to field beans, provided by nine wild pollinator species (A) and by managed honey bees (B). Zero indicates areas lacking pollinator service (minimum service is 0.01 from wild pollinators, 0.002 from managed honey bees). Interval classes are manually defined to the same scale. Blue colour in (B) indicates areas where pollination service cannot be estimated due to missing information on honey bees' presence. Map projection: BNG.

The summed outputs across the nine wild pollinator species, used as a proxy for the total potential pollinator service for field bean, ranged from 0 to 0.43, with a minimum service of 0.01 (Fig. 5(A)): regions close to zero indicate areas where pollinator service is predicted to be low. The predicted pollination service from managed honey bees ranged from 0 (i.e. field bean cells without service from honey bees) to 1, with minimum service of 0.002 (Fig. 5(B)). We also identified areas where pollinator service to field bean cannot be estimated due lack of information on the distribution of managed honey bees (blue regions in Fig. 5(B); File S7: Fig. S7–1 shows the underlying probability of honey bee occurrence).

Taken together, maps in Fig. 5 may be used to qualitatively compare the predicted spatial patterns of potential pollinator service to field bean, based on the current likelihood of occurrence of wild and managed pollinator species in Great Britain. We did not combine the two maps or make quantitative comparisons of patterns across the two groups, due to the different methods used to generate their underlying likelihood of species occurrence. The honey bee index was derived from the estimated maximum forager density based on reported hive location, apiary type and typical foraging distance; the wild pollinator index, instead, was based on a probability of occurrence, which did not take into account number of individuals per species.

## Discussion

In this study we have predicted the current potential distribution of the main crop pollinators of field beans in Great Britain, to derive the potential service provision. Pollinator availability for crop pollination was based on SDMs from species occurrences, rather than on landscape suitability scores from expert knowledge. Potential service provision was assessed for wild and managed pollinators, which to our knowledge has never been done at this scale.

The calibration of the SDMs played an important part in this process making best use of the large species dataset and warranting use of the model outputs as inputs in the pollination service model.

For crops benefiting from insect pollination, we assumed that likelihood of species occurrence can be used as a proxy for potential pollinator service provision, thus implying two main premises: the first one is that the two variables scale proportionally; the second one is that a unit difference in the likelihood of occurrence in one species means the same change in service provision as in a different species.

### Species distribution models

Prior to the modelling work, we tested for sample selection bias within the pollinator records to define the appropriate background: opportunistic records are in fact a great resource to predict species distribution, but they rarely provide a representative sample of the study area. The effects of the choice of background on model predictions are widely documented [19], [41], [51] and therefore it was important that this step was carried out at the start.

In the absence of an independent dataset covering the extent of our study region and the entire spectrum of species, each SDM was built using replication through cross-validation, so that after splitting the occurrence data into groups, models were built and tested using all the groups in turn. An advantage of this method, over using a single partitioning for training and testing, is that it uses all the data for validation, thus making better use of small datasets and minimising the impact of possible outliers.

During model calibration, we used AUC as a threshold-independent measure of model performance. Sole reliance on this method has been criticised [52], [53] as AUC depends on predictive success and not on explanatory value and it is affected by the geographical extent of the model; the latter point is particularly important if AUC is used to compare modelling performance between different species or between models built with different base datasets. In our study, however, we used AUC to compare models based on the same datasets and within species.

The similar AUC between models derived with default settings for feature class and models derived with *Hinge* alone has been observed in at least one other study [42]. In addition, the similar variation in model performance between the 10-fold cross-validation runs, independent of the feature class, indicated comparable stability in their predictions.

Our results on the importance of different predictors indicated a superior discriminatory power within models built with *Hinge* alone, probably due to the greater flexibility of fitted functions when *All* feature classes are allowed. It also became apparent that some of the response curves derived from single-predictor models were too narrowly fitted to the training data when allowing for *All* feature classes (see also [54]), which further supported the choice of using the *Hinge* alone.

The effect of changing the default prevalence to reflect the relative rarity of each species was significant (and positive) only within the bee group, possibly due to their greater variation in number of records. Modifying prevalence has implications for the maximum value predicted by the MaxEnt logistic output [18], noticeable when comparing response curves generated with default and modified prevalence. Since logistic outputs should be interpreted in relation to a temporal and spatial scale appropriate for each species [42], modifying the prevalence allowed us to make the outputs of the SDMs more comparable across species, and to account for their relative differences when evaluating crop pollination service.

The results on predictors' importance highlight *within* and *across* species properties (training vs. testing and mean vs. mode respectively). The *within* species agreement on the predictors' importance between training and testing data suggests that the models are transferable. With climatic predictors being in general the most important ones, this also indicates the possibility of investigating the effects of projected climate changes on the future distribution of wild pollinators. This aspect is of particular interest given the projected shifts in suitable environmental conditions predicted for many taxa including pollinators [55], and the potential phenological mismatch within mutualistic relations, such as plants and pollinators [56], [57]. The significant correlation between the *Mean* and the *Mode* used to rank predictors' importance can be interpreted as a general agreement on their relative importance *across* species.

### Applications to crop pollinators

We adapted Lonsdorf's [8] model to derive pollinator service, using the SDMs derived for the field bean pollinators as inputs. Our choice was motivated by three main reasons: firstly, for the extent of our study area, it would be difficult to rely on expert knowledge to provide landscape suitability scores for pollinators and expert opinion may not be available for poorly known species. Secondly, regularly maintained databases with nation-wide pollinator records offered us the opportunity to rely on actual, albeit opportunistic, sightings. These data have already proven instrumental in detecting changes in species richness across temporal and spatial scales [6], [58]. Thirdly, our approach also accounted for the contribution of managed pollinators, providing the opportunity to compare patterns of pollination service between wild and domesticated pollinators. This is particularly important, given the potentially changing contribution made by both types of crop pollinators in the UK [59]. There is increasing evidence highlighting the importance of wild pollinators to crop production worldwide [60]. However, agricultural intensification and alteration of natural habitats, have shown negative effects on wild pollinator communities [61], [62] and for appropriate mitigation measures to be designed [63], [64], it is crucial to understand how different pollinator species are distributed in space and how this is determined by relationships with their abiotic environment. We believe that the work described here can be used to this end.

Our study has provided predicted PSM for a specific crop, field bean, as a case study to demonstrate how the general approach can be applied to other crops. For application of this method elsewhere we highlight several advantages and further challenges. For instance, since the results are spatially explicit, they can be used to simultaneously investigate the predicted pollinator supply and the underlying extent of crop parcels. This information can help quantify relevant risk factors such as the fraction of crop vulnerable to low pollinator supply. As previously illustrated by the recent work of Lautenbach *et al.* [65] in their map of global pollination benefits, spatially explicit information of this kind can provide a first instrument to prioritize areas where policies aiming at preserving pollination services and mitigating potential pollinator deficits for agricultural crops can be effectively targeted.

Whilst the cross-validation approach used during the SDM allowed us to use the available species occurrences to train and validate the models, testing for significant correlation between the PSM predictions and the pollination service actually provided, would require additional data, which are currently unavailable. In particular, we would need empirical information on pollinator density, flower visitation rate and fruit set for a representative set of crop parcels. Given the extent of the study region, parcels would need to be selected along the gradient of the environmental variables captured by the model, and power analysis would be needed to determine how may parcel replicates would be necessary to achieve the desired level of confidence. An additional difficulty relates to the scale (resolution) of the current model, which is suitable for country-wide and local scale patterns, but may be too coarse to draw correlations with what is observed at the crop parcel scale. We recommend that future applications of our method consider building models with species and environmental layers matching the spatial scale of the field work, thereby allowing direct testing of predictions. The empirical information being collected in different agricultural systems worldwide has already proven instrumental for drawing general patterns, such as the relative importance of wild pollinators vs. managed pollinators for enhancing fruit set (e.g. [60] and references therein). A number of studies funded under the UK Insect Pollinator Initiative, may provide the information needed towards a first validation step over the next few years.

### Conclusion and Next Steps

The primary interest of our study was to show how the Lonsdorf *et al.* [8] pollination service model can be integrated with the MaxEnt species distribution model [13] to predict geographical patterns of pollination service to crops. We chose these two models since they both have peer-reviewed track records of successful applications in their respective fields but, to our knowledge, they have never been used in combination. The two main elements of novelty in our study are the use of pollinator records rather than expert knowledge to predict wild pollinator occurrence, and the inclusion of managed pollinator data. This approach allowed us to map the relative contribution of each pollinator group, and also identify areas potentially vulnerable to low service provision. Thus the outputs can help direct local scale mitigation measures, such as agri-environment scheme options. Despite the difficulties common to proxy-based approaches [66] the method we have proposed is sufficiently flexible to incorporate different environmental variables of biological relevance, which may be available for other geographic regions, useful to refine predictions, or relevant when the models are applied to smaller spatial extents. The last point should be of particular interest to studies at the field parcel scale, where detailed information of landscape elements may be collected and used to build the models. The possibility to correlate relative scores and proxies to empirical data is likely to provide relevant information for both the SDM and the PSM: for instance, using information on farm management and landscape composition and configuration, Kennedy *et al.* [67] have assessed the strength of the correlation between different predictors of bee abundance and richness and empirical data collected in 39 crop systems across the globe.

Looking ahead, the inclusion of local pollinator abundance and of the pollination effectiveness of different pollinators are arguably the most urgent next challenges we need to face to help to translate the relative suitability scores into units of crop pollination service and ultimately yield. Service provision, in fact, results from species' efficiency and local abundance.

We have used field beans as test case, but the method we have illustrated can be applied to other crops, provided that their distribution and main pollinators are known. In addition, this approach can be extended to investigate the projected effects of climate change on pollination services. To do that, it would require predictive SDMs for both the crop of interest and its pollinators, to reveal any compositional change in the pollinator community, as well as any potential geographical mismatch between crop and pollinators.

## Supporting Information

File S1 **Figure S1–1: Number of records from Web of Knowledge for applications of MaxEnt in species distribution models.** Search criteria: Topic  =  “Maxent” AND “Species distribution”; Years  =  from 2006 to 2012; access date: 28/08/2012.
(PDF)

File S2 **Table S2–1: Species selected for model calibration.** Sample size equals to the number of occupied 1 km^2^ grid cells, which becomes the area occupied by a species solely based on existing records; quartile distance is the longest distance between all pairwise records for a particular species within its 3^rd^ quartile. **Table S2–2: Pearson's correlation between selected topographic and bio-climatic variables.** Predictors are defined in the main text.
(PDF)

File S3 **Figure S3–1: Number of species within each class of modified prevalence (τ), for bees (grey) and hoverflies (black). Table S3–1: Revised values of τ for species used during model calibration.** **Figure 3** **–2.1 Single response curves for** ***Andrena niveata*** **with default and modified prevalence.** Response of *A. niveata* to mean temperature of the driest quarter, with default (0.5, panel A) and modified prevalence (0.1, panel B). Modifying the prevalence changes the maximum probability of presence from ∼0.65 to ∼0.17. The response curves are based on a (MaxEnt) model created using only the focal predictor. The curves show the mean response of the 10 runs (red) and the mean +/− one standard deviation (blue). **Figure 3** **–2.2: Single response curves for** ***Rhingia rostrata*** **with default and modified prevalence.** Response of *R. rostrata* to percentage of arable land, with default (0.5, panel A) and modified (0.3, panel B) prevalence. The maximum predicted probability of presence changes from ∼0.55 to ∼0.35. The response curves are based on a (MaxEnt) model created using only the focal predictor. The curves show the mean response of the 10 runs (red) and the mean +/− one standard deviation (blue).
(PDF)

File S4 **Table S4–1: Results of the mixed model evaluating the influence of different model settings on the model performance**. Model performance from the AUC of test data, for Bee and Hoverfly. Fixed effects only are shown here. A star (*) indicates that modified values of prevalence were used. **Table S4–2: Results of the mixed model evaluating the influence of different model settings on the variability of the model performance.** Model performance from the Standard Deviation of the AUC of test data (from the 10 cross-validations), for Bee and Hoverfly. Fixed effects only are shown here. A star (*) indicates that modified values of prevalence were used. **Table S4–3: Results on the discriminatory ability of models built with different feature classes and prevalence, from generalized linear models.** The importance of different predictors was better discriminated in models built using *Hinge* feature class alone. All*  =  *All* features classes allowed, with modified prevalence. Hinge  =  only *Hinge* feature class, with default prevalence. Hinge*  =  only *Hinge* feature class, with modified prevalence. **Figure S4–1.1: Single response curves for** ***Andrena barbilabris*** **with default feature class and hinge only.** Response of *A. barbilabris* (prevalence  = 0.5) to the mean temperature of driest quarter as modelled by default settings for feature class (A) and hinge only (B). See main text for explanations. **Figure S4–1.2: Single response curves for** ***Syrphus ribesii*** **with default feature class and hinge only.** Response of *S. ribesii* (prevalence  = 0.4) to the temperature annual range as modelled by default settings for feature class (A) and hinge only (B). See main text for explanations. **Figure S4–1.3: Single response curves for** ***Bombus muscorum*** **with default feature class and hinge only.** Response of *B. muscorum* (prevalence  = 0.3) to the coefficient of variation of precipitation seasonality, as modelled by default settings for feature class (A) and hinge only (B). See main text for explanations. **Figure S4–1.4: Single response curves for** ***Osmia rufa*** **with default feature class and hinge only.** Response of *Osmia rufa* (prevalence  = 0.2) to the mean temperature of coldest quarter, as modelled by default settings for feature class (A) and hinge only (B). See main text for explanations.
(PDF)

File S5 **Figure S5–1.1: Predicted probability of occurrence of** ***Andrena labiata*** **along the temperature annual range.** Figure S5–1.2: Predicted probability of occurrence of *Andrena minutuloides* along the precipitation seasonality. Figure S5–1.3: Predicted probability of occurrence of *Halictus rubicundus* along the precipitation of the coldest quarter. Figure S5–1.4: Predicted probability of occurrence of *Megachile maritima* along the mean temperature of the coldest quarter. Figure S5–2: Rank correlation within training and testing data, for predictors *Mean* and *Mode*. **Spearman's rank correlations: Mean (open squares and dashed line): ρ = 0.974; Mode (filled circles and solid line): ρ = 0.944. Both correlations are significant, based on 999 bootstrap replicates (Fig. S5–4 panel A and B).** Figure S5–3: Rank correlation between *Mean* and *Mode*, for the pooled set of training and test models across species. **Spearman's rank correlation: ρ = 0.940, significant based on 999 bootstrap replicates (Fig. S5–4 panel C).** Figure S5–4: Distributions of bootstrap and observed Spearman**'**s rank correlations. **A: correlation of predictors' Mean between training and testing phase; B: correlation of predictors'** ***Mode*** **between training and testing phase; C: correlation between** ***Mean*** **and** ***Mode*** **for the pooled set of training and testing data. In all three cases the observed correlation are significantly greater than those generated from 999 bootstrap replicates.**
(PDF)

File S6 **Figure S6–1: Probability of occurrence (A) and potential pollinator service (B) for** ***A. labialis*** **. Figure S6–2: Probability of occurrence (A) and potential pollinator service (B) for** ***A. wilkella*** **. Figure S6–3: Probability of occurrence (A) and potential pollinator service (B) for** ***B. hortorum*** **. Figure S6–4: Probability of occurrence (A) and potential pollinator service (B) for** ***B. lapidarius*** **. Figure S6–5: Probability of occurrence (A) and potential pollinator service (B) for** ***B. lucorum*** **. Figure S6–6: Probability of occurrence (A) and potential pollinator service (B) for** ***B. muscorum*** **. Figure S6–7: Probability of occurrence (A) and potential pollinator service (B) for** ***B. terrestris*** **. Figure S6–8: Probability of occurrence (A) and potential pollinator service (B) for** ***O. rufa*** **.**
(PDF)

File S7 **Figure S7–1: Probability of occurrence of managed honey bees.** The original density of foragers was linearly rescaled to 0–1 and the 0–1 and the 5^th^ percentile threshold was adopted to distinguish absence from presence (corresponding to a 0.001 probability of occurrence). Map projection: British National Grid.
(PDF)
