# Supplementary material for: Species Distribution Models for Crop Pollination: A Modelling Framework Applied to Great Britain
Source: PLoS One. 2013 Oct 14;8(10):e76308. doi: 10.1371/journal.pone.0076308 (PMC3796555; doi:10.1371/journal.pone.0076308)
Supplement: File S1 — Figure S1–1: Number of records from Web of Knowledge for applications of MaxEnt in species distribution models. Search criteria: Topic = “Maxent” AND “Species distribution”; Years = from 2006 to 2012; access date: 28/08/2012. (PDF) [file pone.0076308.s001.pdf]

FILE S1: INTRODUCTION

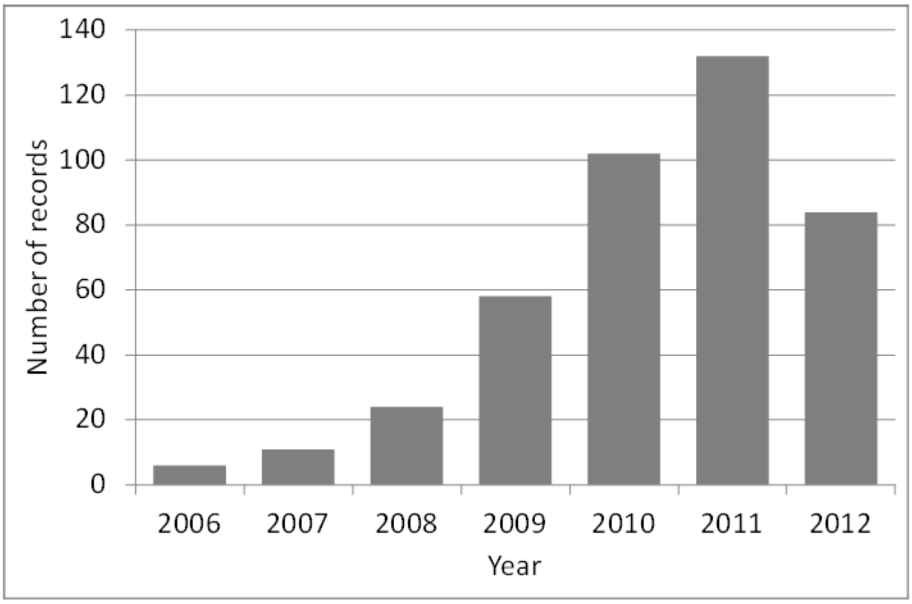

Figure S1-1: Number of records from Web of Knowledge for applications of MaxEnt in species distribution models.

Search criteria: Topic = “Maxent” AND “Species distribution”; Years = from 2006 to 2012; access date: 28/08/2012.
