# Supplementary material for: Species Distribution Models for Crop Pollination: A Modelling Framework Applied to Great Britain
Source: PLoS One. 2013 Oct 14;8(10):e76308. doi: 10.1371/journal.pone.0076308 (PMC3796555; doi:10.1371/journal.pone.0076308)
Supplement: File S2 — Table S2–1: Species selected for model calibration. Sample size equals to the number of occupied 1 km2 grid cells, which becomes the area occupied by a species solely based on existing records; quartile distance is the longest distance between all pairwise records for a particular species within its 3rd quartile. Table S2–2: Pearson's correlation between selected topographic and bio-climatic variables. Predictors are defined in the main text. (PDF) [file pone.0076308.s002.pdf]

FILE S2: DATASETS

Table S2-1: Species selected for model calibration.

Sample size equals to the number of occupied 1 km^2 grid cells, which becomes the area occupied by a species solely based on existing records; quartile distance is the longest distance between all pairwise records for a particular species within its 3rd quartile.

Bees

Species name	Sample size	Quartile distance (km)
Andrena barbilabris	228	340.4
A. labialis	184	185.8
A. labiata	183	163.2
A. minutuloides	104	97.7
A. niveata	12	63.8
Anthophora plumipes	447	198.3
Bombus muscorum	599	763.9
B. pascuorum	4254	506.9
B. terrestris	2628	301.8
Halictus rubicundus	502	452.7
Lasioglossum brevicorne	45	139.6
L. fratellum	144	439.9
L. malachurum	536	140.0
L. nitidiusculum	30	382.7
L. rufitarse	72	435.7
L. semilucens	13	115.2
L. villosulum	617	280.2
L. xanthopus	66	164.2
Megachile centuncularis	245	326.7
M. maritima	106	299.7
Osmia bicolor	186	141.4
O. rufa	1104	241.3

Hoverflies

Species name	Sample size	Quartile distance (km)
Episyrphus balteatus	4105	268.3
Eristalis horticola	583	312.6
E. tenax	2846	269.8
Rhingia campestris	2196	278.2
R. rostrata	233	215.4
Syrphus ribesii	2001	257.9

Table S2-2: Pearson's correlation between selected topographic and bio-climatic variables.

To minimize multicollinearity [1] within the original set of bio-climatic and topographic variables (19 and 4 respectively), we applied Jolliffe's Principal Component Analysis with the rejection method "B2" [2], removing variables associated to components with eigenvalue < λ_0 (usually $0.69 \leq \lambda_0 \leq 0.74$). Predictors are defined in the main text.

	**RainSeasCV**	**RainCQ**	**Isoth**	**TAR**	**MTDQ**	**MTCQ**	**AspEW**
**RainSeasCV**							
**RainCQ**	0.6						
**Isoth**	0.00	0.08					
**TAR**	-0.34	-0.60	0.37				
**MTDQ**	0.46	0.33	0.16	-0.24			
**MTCQ**	0.15	-0.26	-0.10	-0.01	0.20		
**AspEW**	-0.07	-0.04	0.01	0.01	-0.09	-0.02	
**AspNS**	-0.03	-0.07	-0.01	0.09	-0.03	-0.03	-0.02

REFERENCES

1. Guisan A, Thuiller W (2005) Predicting species distribution: offering more than simple habitat models. Ecol Lett 8: 993-1009.
2. Jolliffe IT (1973) Discarding Variables in a Principal Component Analysis, II: Real Data. Applied Statistics 22: 21-31.
