# Supplementary material for: Species Distribution Models for Crop Pollination: A Modelling Framework Applied to Great Britain
Source: PLoS One. 2013 Oct 14;8(10):e76308. doi: 10.1371/journal.pone.0076308 (PMC3796555; doi:10.1371/journal.pone.0076308)
Supplement: File S3 — Figure S3–1: Number of species within each class of modified prevalence (τ), for bees (grey) and hoverflies (black). Table S3–1: Revised values of τ for species used during model calibration. Figure 3 –2.1 Single response curves for Andrena niveata with default and modified prevalence. Response of A. niveata to mean temperature of the driest quarter, with default (0.5, panel A) and modified prevalence (0.1, panel B). Modifying the prevalence changes the maximum probability of presence from ∼0.65 to ∼0.17. The response curves are based on a (MaxEnt) model created using only the focal predictor. The curves show the mean response of the 10 runs (red) and the mean +/− one standard deviation (blue). Figure 3 –2.2: Single response curves for Rhingia rostrata with default and modified prevalence. Response of R. rostrata to percentage of arable land, with default (0.5, panel A) and modified (0.3, panel B) prevalence. The maximum predicted probability of presence changes from ∼0.55 to ∼0.35. The response curves are based on a (MaxEnt) model created using only the focal predictor. The curves show the mean response of the 10 runs (red) and the mean +/− one standard deviation (blue). (PDF) [file pone.0076308.s003.pdf]

FILE S3: PREVALENCE

To determine prevalence, the proportion of occupied sites, MaxEnt treats the log of the raw output as logit score: the logit score is used to calibrate the intercept so that the probability of presence at sites with “typical” conditions is the parameter τ. Knowing τ provides a solution to obtaining prevalence and in absence of other knowledge, τ is arbitrarily set to 0.5. The probability of presence, however, is defined over specific spatial and temporal scales [1], which should be taken into account particularly when working with pools of species with different degrees of commonality / rarity. In our study we run part of the models with modified values of τ, based on the range of commonality observed within the data.

The information we used to establish new values for τ was the following:

- Temporal scale: 11 years: from 2000 to present, with the exclusion of 2011 as only 1 record was present in the database;
- Spatial scale [*a*]: number of occupied cells at the 1 x 1 km grain (=”occupied area”), for each species;
- “Rarity” [*b*]: total number of records at any grain, from 2000 to present, for each species;
- Years with records [*c*]: number of years with non-zero observations, for each species.

Based on these definitions, we derived for each species:

- $d = b/a$, the average number of records per km^2 (“species’ density”)
- f = $c/11$, a correction factor for species having 0 (zero) records at any year;
- $\tau_{temp} = \log(d)*f$, to weigh the obtained density by the ratio f;

The revised τ for each species was obtained as:

$\tau_{rev} = (\tau_{temp} * 0.5)/ \max(\tau_{temp})$

Where 0.5 = default τ; $\max(\tau_{temp})$ = maximum value of τ_{temp} observed within the data.

Figure S3-1 shows the number of species for each revised τ. Their identity is shown in Table S3-1.

As an example of the effects of modifying prevalence, Figs S3-2.1 and S3-2.2 show some of the species’ response curves in models built with one predictor in turn, with default (0.5) and modified prevalence. For all curves shown in Figs S3-2.1 and S3-2.2, the red line indicates the average response from the 10-fold cross-validation runs, whilst the blue area shows one standard deviation from the mean. Predictors are defined in the main text.

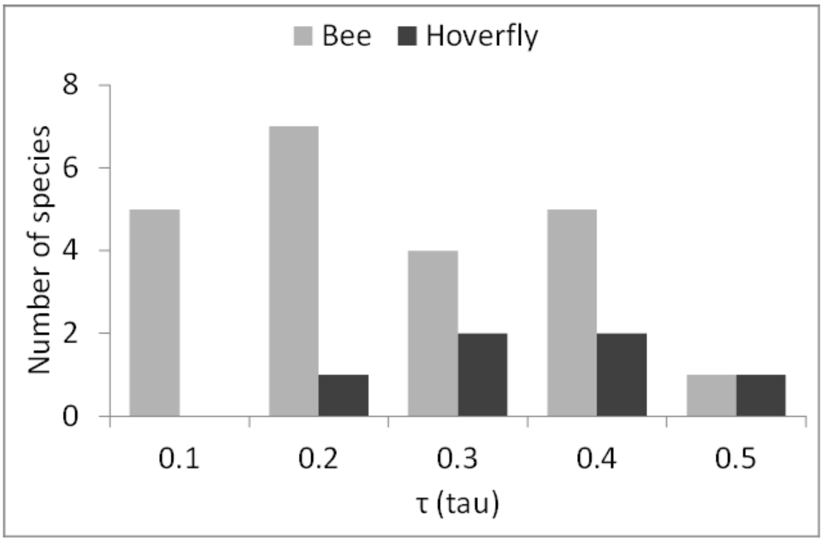

Figure S3-1: Number of species within each class of modified prevalence (τ), for bees (grey) and hoverflies (black).

Table S3-1: Revised values of τ for species used during model calibration.

τ	Bees	Hoverflies
0.1	*Andrena niveata*<br>*Lasioglossum fratellum*<br>*L. nitidiusculum*<br>*L. semilucens*<br>*L. xanthopus*	None
0.2	*Lasioglossum brevicorne*<br>*L. malachurum*<br>*L. rufitarse*<br>*L. villosulum*<br>*Megachile centuncularis*<br>*M. maritima*<br>*Osmia rufa*	*Eristalis horticola*
0.3	*Andrena labialis*<br>*A. labiata*<br>*Bombus muscorum*<br>*Halictus rubicundus*	*Rhingia campestris*<br>*R. rostrata*
0.4	*Andrena minutuloides*<br>*Anthophora plumipes*<br>*Bombus pascuorum*<br>*B. terrestris*<br>*Osmia bicolor*	*Eristalis tenax*<br>*Syrphus ribesii*
0.5	*Andrena barbilabris*	*Episyrphus balteatus*

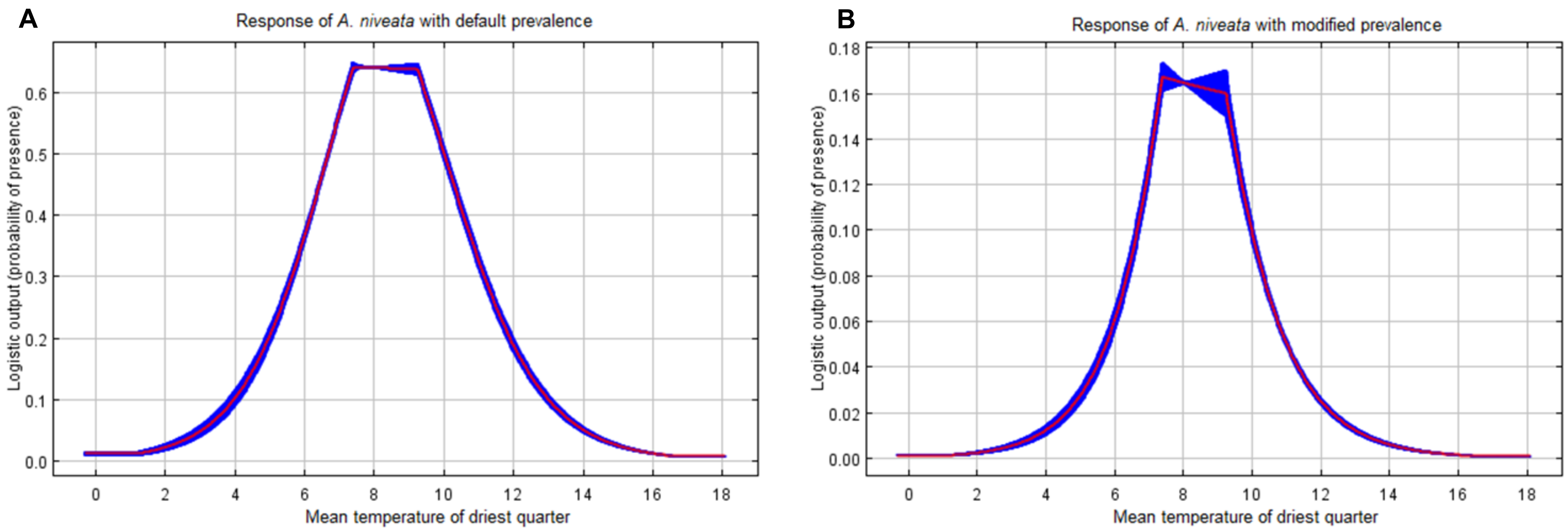

Figure S3-2.1: Single response curves for *Andrena niveata* with default and modified prevalence.

Response of *A. niveata* to mean temperature of the driest quarter, with default (0.5, panel A) and modified prevalence (0.1, panel B). Modifying the prevalence changes the maximum probability of presence from ~ 0.65 to ~ 0.17. The response curves are based on a (MaxEnt) model created using only the focal predictor. The curves show the mean response of the 10 runs (red) and the mean +/- one standard deviation (blue).

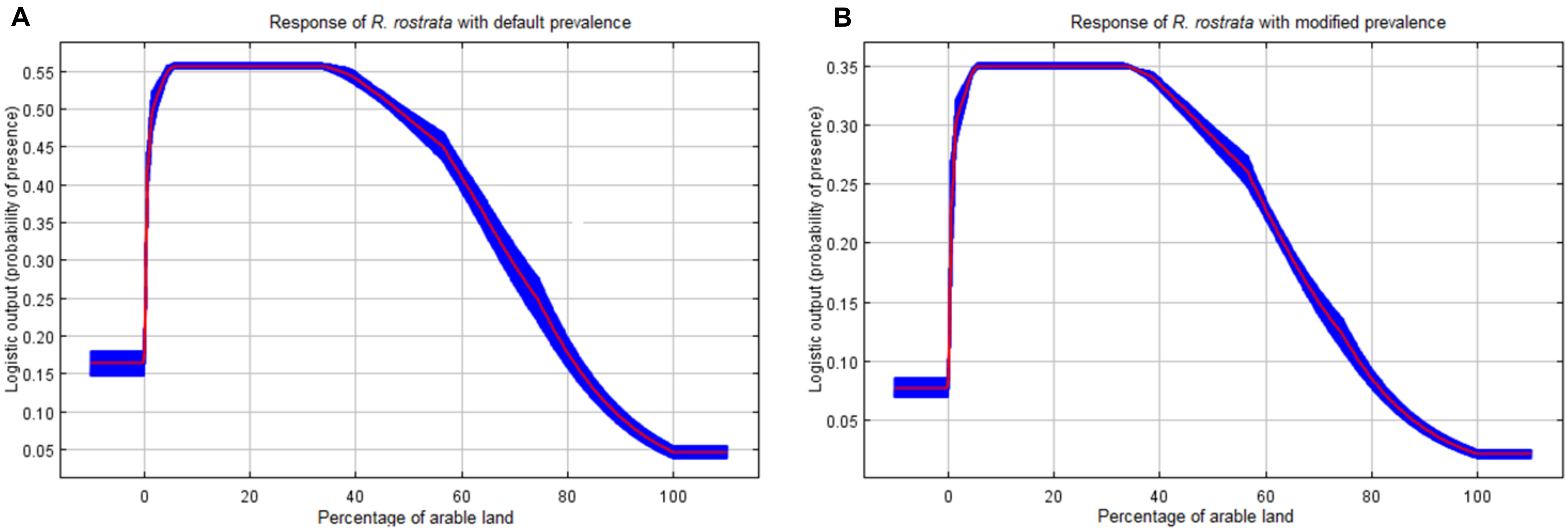

Figure S3-2.2: Single response curves for *Rhingia rostrata* with default and modified prevalence.

Response of *R. rostrata* to percentage of arable land, with default (0.5, panel A) and modified (0.3, panel B) prevalence. The maximum predicted probability of presence changes from ~ 0.55 to ~ 0.35. The response curves are based on a (MaxEnt) model created using only the focal predictor. The curves show the mean response of the 10 runs (red) and the mean +/- one standard deviation (blue).

REFERENCES

1. Elith J, Phillips SJ, Hastie T, Dudík M, Chee YE, et al. (2011) A statistical explanation of MaxEnt for ecologists. Divers Distrib 17: 43-57.
