# Supplementary material for: Species Distribution Models for Crop Pollination: A Modelling Framework Applied to Great Britain
Source: PLoS One. 2013 Oct 14;8(10):e76308. doi: 10.1371/journal.pone.0076308 (PMC3796555; doi:10.1371/journal.pone.0076308)
Supplement: File S4 — Table S4–1: Results of the mixed model evaluating the influence of different model settings on the model performance. Model performance from the AUC of test data, for Bee and Hoverfly. Fixed effects only are shown here. A star (*) indicates that modified values of prevalence were used. Table S4–2: Results of the mixed model evaluating the influence of different model settings on the variability of the model performance. Model performance from the Standard Deviation of the AUC of test data (from the 10 cross-validations), for Bee and Hoverfly. Fixed effects only are shown here. A star (*) indicates that modified values of prevalence were used. Table S4–3: Results on the discriminatory ability of models built with different feature classes and prevalence, from generalized linear models. The importance of different predictors was better discriminated in models built using Hinge feature class alone. All* = All features classes allowed, with modified prevalence. Hinge = only Hinge feature class, with default prevalence. Hinge* = only Hinge feature class, with modified prevalence. Figure S4–1.1: Single response curves for Andrena barbilabris with default feature class and hinge only. Response of A. barbilabris (prevalence = 0.5) to the mean temperature of driest quarter as modelled by default settings for feature class (A) and hinge only (B). See main text for explanations. Figure S4–1.2: Single response curves for Syrphus ribesii with default feature class and hinge only. Response of S. ribesii (prevalence = 0.4) to the temperature annual range as modelled by default settings for feature class (A) and hinge only (B). See main text for explanations. Figure S4–1.3: Single response curves for Bombus muscorum with default feature class and hinge only. Response of B. muscorum (prevalence = 0.3) to the coefficient of variation of precipitation seasonality, as modelled by default settings for feature class (A) and hinge only (B). See main text for explanations. Figure S4–1.4: S [file pone.0076308.s004.pdf]

# FILE S4: MODEL CALIBRATION

*<u>Test 1:</u> Do different combinations of feature classes and prevalence affect model performance as measured by the AUC?*

Model performance was measured as average (of 10 cross-validations) AUC of test data, for Bee and Hoverfly, using mixed-effects models, with species' identity as random factors. The results are summarised in Table S4-1 and show that changing feature class and prevalence did not significantly affect model performance.

**Table S4-1: Results of the mixed model evaluating the influence of different model settings on the model performance.**

Model performance from the AUC of test data, for Bee and Hoverfly. Fixed effects only are shown here. A star (*) indicates that modified values of prevalence were used.

| | Bee | | | | | Hoverfly | | | | |
|---|---|---|---|---|---|---|---|---|---|---|
| | Estimate | SE | DF | t | P | Estimate | SE | DF | t | P |
| Intercept | 0.794 | 0.018 | 61 | 43.238 | < 0.001 | 0.703 | 0.035 | 13 | 20.199 | < 0.001 |
| All* | 0.000 | 0.004 | 61 | 0.023 | 0.982 | 0.003 | 0.002 | 13 | 1.417 | 0.180 |
| Hinge | 0.003 | 0.004 | 61 | 0.789 | 0.433 | 0.000 | 0.002 | 13 | 0.000 | 1.000 |
| Hinge* | 0.003 | 0.004 | 61 | 0.745 | 0.459 | 0.001 | 0.002 | 13 | 0.626 | 0.542 |

*<u>Test 2</u>: Does the variability in model performance (Standard Deviation of the AUC) vary significantly between different combinations of feature classes and prevalence?*

Model performance was measured as Standard Deviation of the AUC of test data (from the 10 cross-validations), for Bee and Hoverfly, with species' identity as random factors. The results are summarised in Table S4-2 and show that, in general, changing the feature class and prevalence did not significantly affect the variability in model performance. For Hoverfly, however, changing the default prevalence for feature class "All" increased the Standard Deviation of the AUC.

**Table S4-2: Results of the mixed model evaluating the influence of different model settings on the variability of the model performance.**

Model performance from the Standard Deviation of the AUC of test data (from the 10 cross-validations), for Bee and Hoverfly. Fixed effects only are shown here. A star (*) indicates that modified values of prevalence were used.

| | Bee | | | | | Hoverfly | | | | |
|---|---|---|---|---|---|---|---|---|---|---|
| | Estimate | SE | DF | t | P | Estimate | SE | DF | t | P |
| Intercept | -3.123 | 0.169 | 61 | -18.520 | < 0.001 | -4.078 | 0.121 | 13 | -33.716 | < 0.001 |
| All* | 0.034 | 0.079 | 61 | 0.427 | 0.671 | 0.223 | 0.097 | 13 | 2.296 | 0.039 |
| Hinge | -0.088 | 0.078 | 61 | -1.140 | 0.259 | -0.017 | 0.091 | 13 | -0.184 | 0.857 |
| Hinge* | -0.075 | 0.079 | 61 | -0.950 | 0.346 | 0.170 | 0.097 | 13 | 1.753 | 0.103 |

*<u>Test 3:</u> Does the standard deviation (SD) of the Permutation importance (%) between predictors and background vary significantly between different combinations of feature classes and prevalence?*

The ability to discriminate the importance of different predictors was measured as the SD of the Permutation importance (%). The results are summarised in Table S4-3 and show that the importance of the different predictors was more distinct in models built using Hinge feature class alone.

**Table S4-3: Results on the discriminatory ability of models built with different feature classes and prevalence, from generalised linear models.**

The importance of different predictors was better discriminated in models built using *Hinge* feature class alone. **All*** = *All* features classes allowed, with modified prevalence. **Hinge** = only *Hinge* feature class, with default prevalence. **Hinge*** = only *Hinge* feature class, with modified prevalence.

| | Bee | | | | Hoverfly | | | |
|---|---|---|---|---|---|---|---|---|
| | Estimate | SE | t | P | Estimate | SE | t | P |
| Intercept | 1.788 | 0.064 | 28.060 | < 0.001 | 1.664 | 0.043 | 38.476 | < 0.001 |
| All* | 0.012 | 0.091 | 0.126 | 0.900 | -0.034 | 0.064 | -0.524 | 0.607 |
| Hinge | 0.317 | 0.090 | 3.518 | 0.001 | 0.343 | 0.061 | 5.615 | < 0.001 |
| Hinge* | 0.345 | 0.091 | 3.781 | < 0.001 | 0.326 | 0.064 | 5.077 | < 0.001 |

We further inspected a random sample of species responses in models built with only one predictor in turn: response curves generated with default settings for feature class (where all types may be used) were more complex than those obtained with *Hinge* alone; in some cases this complexity suggested a too narrowly fit response. Examples of these curves are provided in Figs S4-1.1 to S4-1.4, for species with different values of prevalence. In each figure, the red line indicates the average response from 10 cross-validation runs, whilst the blue area shows one standard deviation from the mean.

Based on these patterns and on the results obtained on model performance (AUC and $AUC_{SD}$) and Predictors Importance (SD of the Permutation Importance %) we chose to adopt *Hinge* feature class alone (with modified prevalence) to derive SDMs for the set of pollinators relevant to British crops.

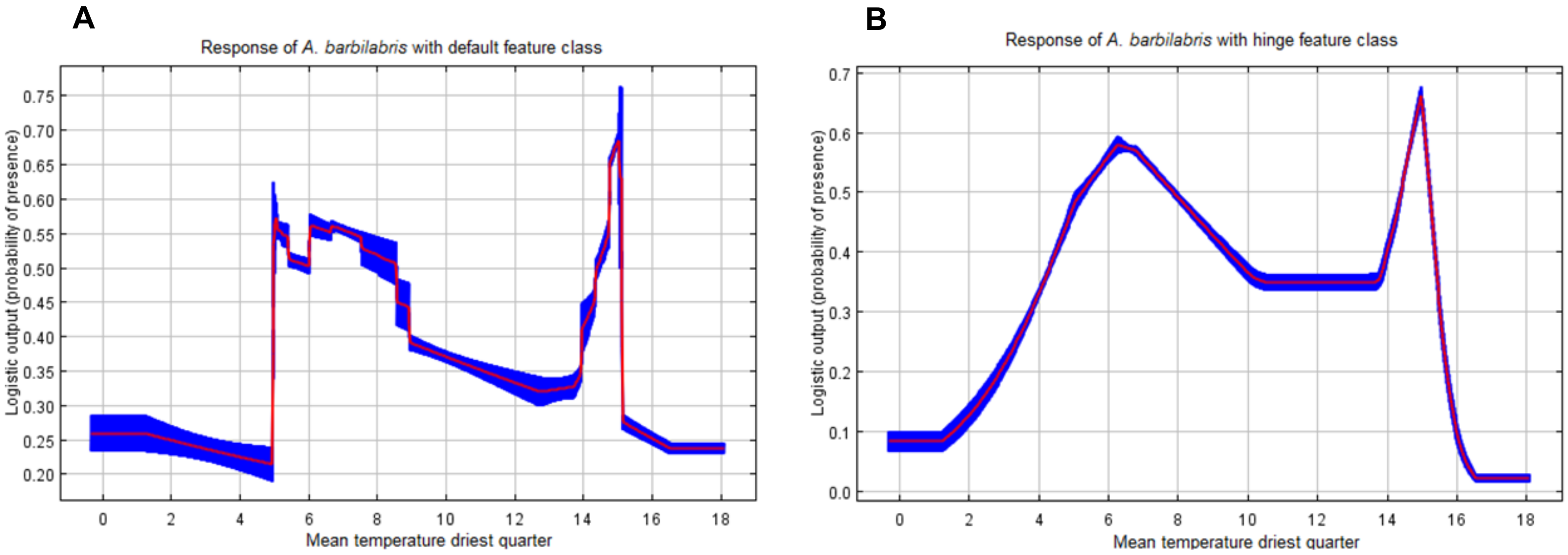


**Figure S4-1.1: Single response curves for *Andrena barbilabris* with default feature class and hinge only.**

Response of *A. barbilabris* (prevalence = 0.5) to the mean temperature of driest quarter as modelled by default settings for feature class (A) and hinge only (B). See main text for explanations.

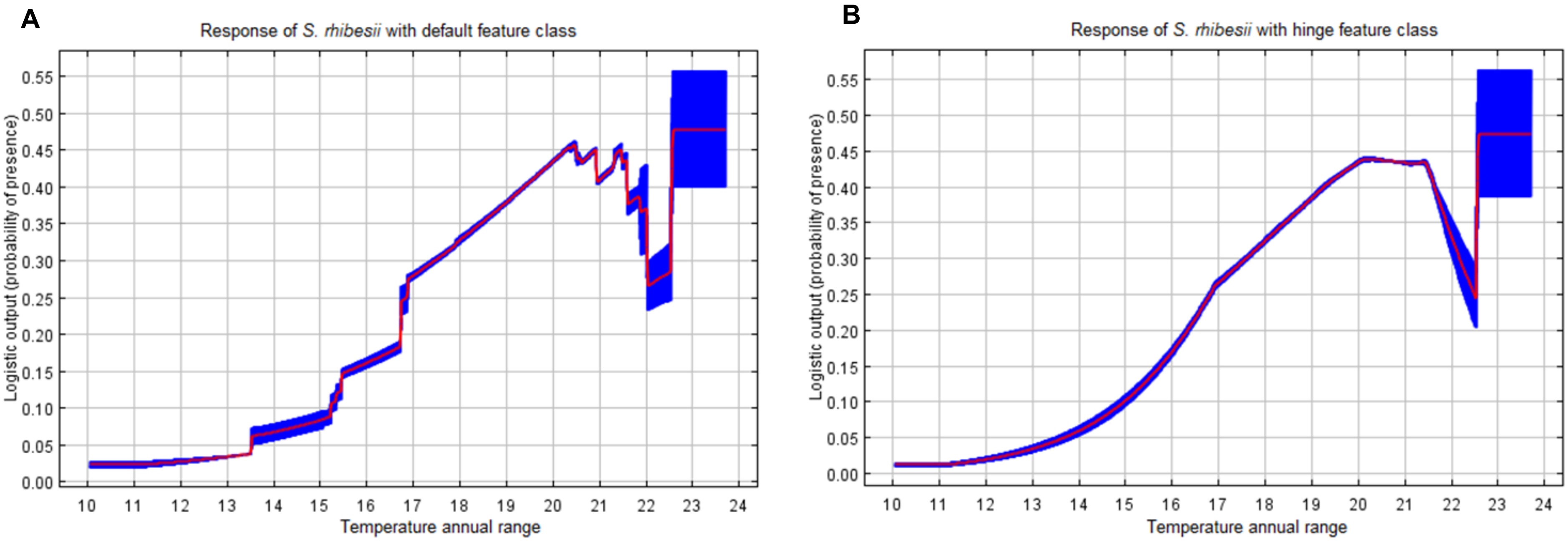


**Figure S4-1.2: Single response curves for *Syrphus ribesii* with default feature class and hinge only.**

Response of *S. ribesii* (prevalence = 0.4) to the temperature annual range as modelled by default settings for feature class (A) and hinge only (B). See main text for explanations.

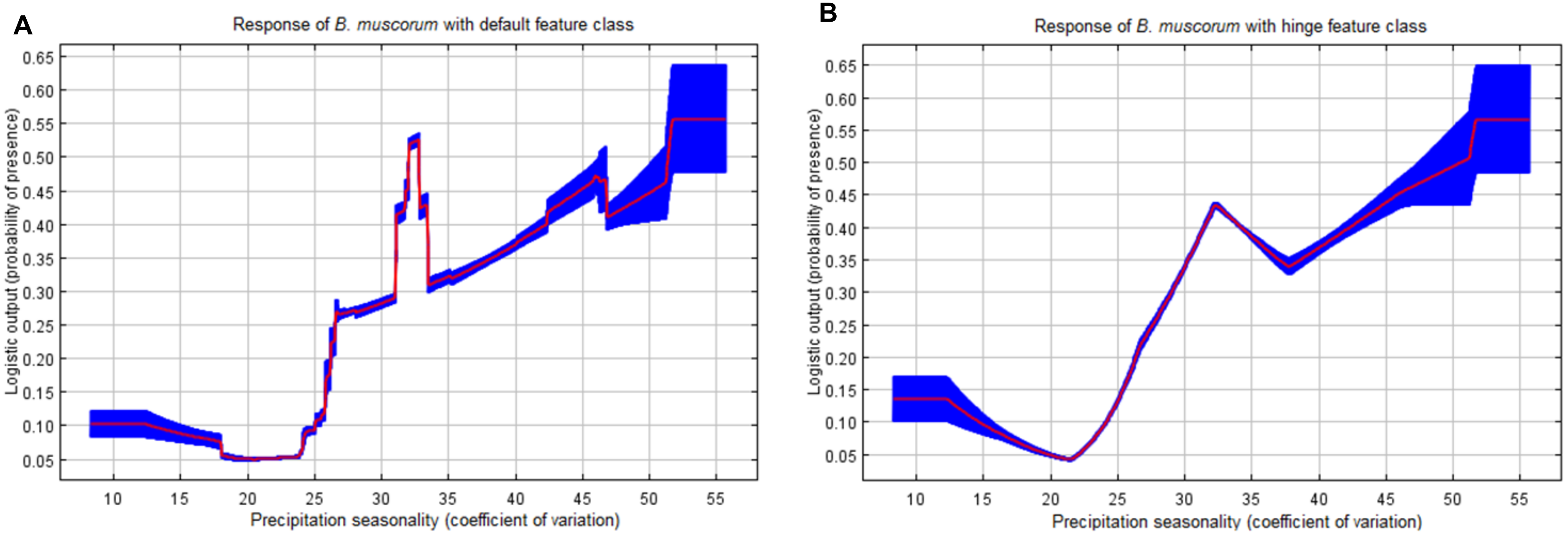


**Figure S4-1.3: Single response curves for *Bombus muscorum* with default feature class and hinge only.**

Response of *B. muscorum* (prevalence = 0.3) to the coefficient of variation of precipitation seasonality, as modelled by default settings for feature class (A) and hinge only (B). See main text for explanations.

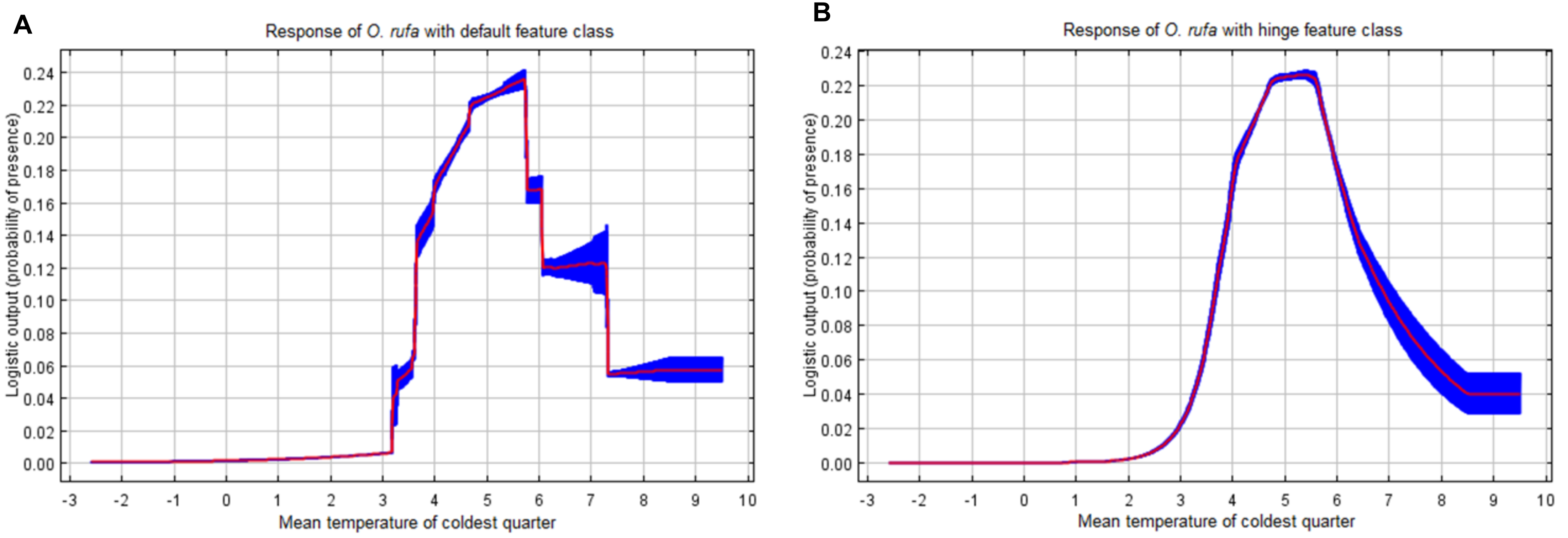


**Figure S4-1.4: Single response curves for *Osmia rufa* with default feature class and hinge only.**

Response of *Osmia rufa* (prevalence = 0.2) to the mean temperature of coldest quarter, as modelled by default settings for feature class (A) and hinge only (B). See main text for explanations.
