# Supplementary material for: Species Distribution Models for Crop Pollination: A Modelling Framework Applied to Great Britain
Source: PLoS One. 2013 Oct 14;8(10):e76308. doi: 10.1371/journal.pone.0076308 (PMC3796555; doi:10.1371/journal.pone.0076308)
Supplement: File S5 — Figure S5–1.1: Predicted probability of occurrence of Andrena labiata along the temperature annual range. Figure S5–1.2: Predicted probability of occurrence of Andrena minutuloides along the precipitation seasonality. Figure S5–1.3: Predicted probability of occurrence of Halictus rubicundus along the precipitation of the coldest quarter. Figure S5–1.4: Predicted probability of occurrence of Megachile maritima along the mean temperature of the coldest quarter. Figure S5–2: Rank correlation within training and testing data, for predictors Mean and Mode. Spearman's rank correlations: Mean (open squares and dashed line): ρ = 0.974; Mode (filled circles and solid line): ρ = 0.944. Both correlations are significant, based on 999 bootstrap replicates (Fig. S5–4 panel A and B). Figure S5–3: Rank correlation between Mean and Mode, for the pooled set of training and test models across species. Spearman's rank correlation: ρ = 0.940, significant based on 999 bootstrap replicates (Fig. S5–4 panel C). Figure S5–4: Distributions of bootstrap and observed Spearman's rank correlations. A: correlation of predictors' Mean between training and testing phase; B: correlation of predictors' Mode between training and testing phase; C: correlation between Mean and Mode for the pooled set of training and testing data. In all three cases the observed correlation are significantly greater than those generated from 999 bootstrap replicates. (PDF) [file pone.0076308.s005.pdf]

FILE S5: IMPORTANCE OF PREDICTORS

Figures from S5-1.1 to S5-1.4 show the single response curves to the four most important predictors, for the four species where one of these predictors scored the greatest importance. Figures from S5-2 to S5-4 show the correlation of the *Mean* and *Mode* of the predictors' importance, and their significance.

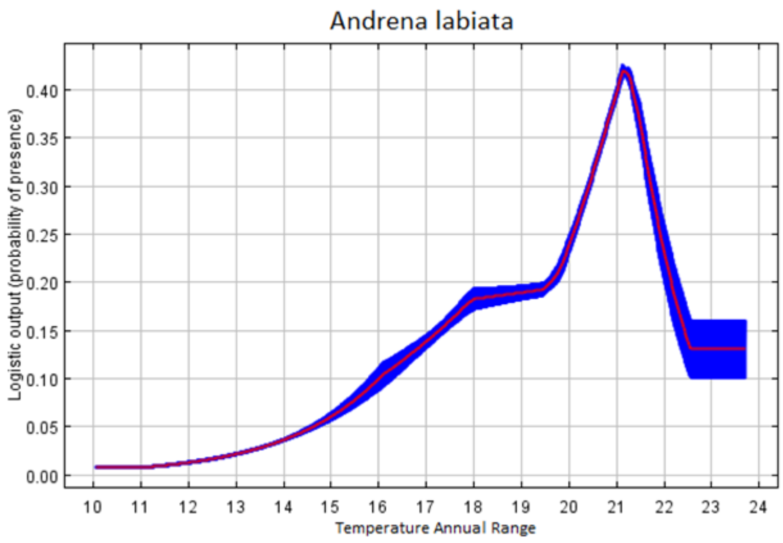

Figure S5-1.1: Predicted probability of occurrence of *Andrena labiata* along the temperature annual range.

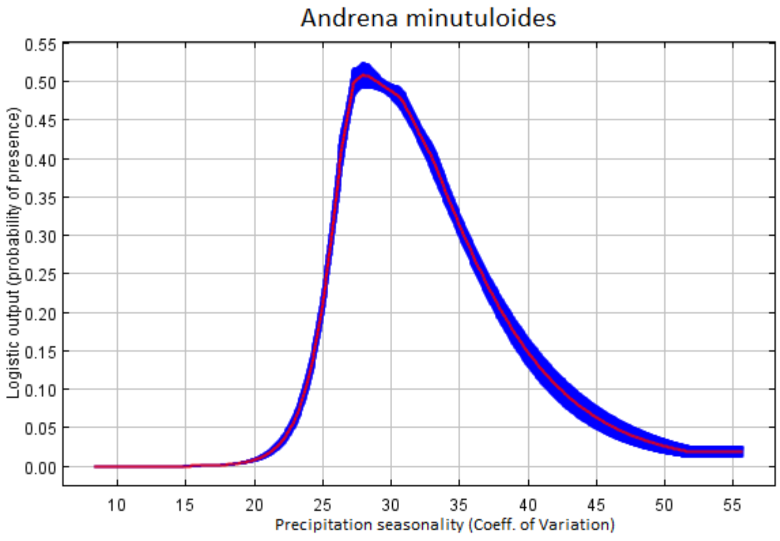

Figure S5-1.2: Predicted probability of occurrence of *Andrena minutuloides* along the precipitation seasonality.

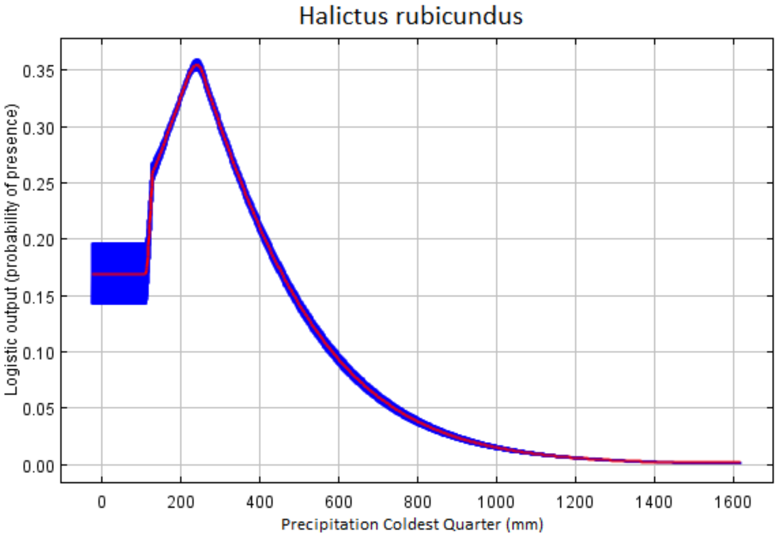

Figure S5-1.3: Predicted probability of occurrence of *Halictus rubicundus* along the precipitation of the coldest quarter.

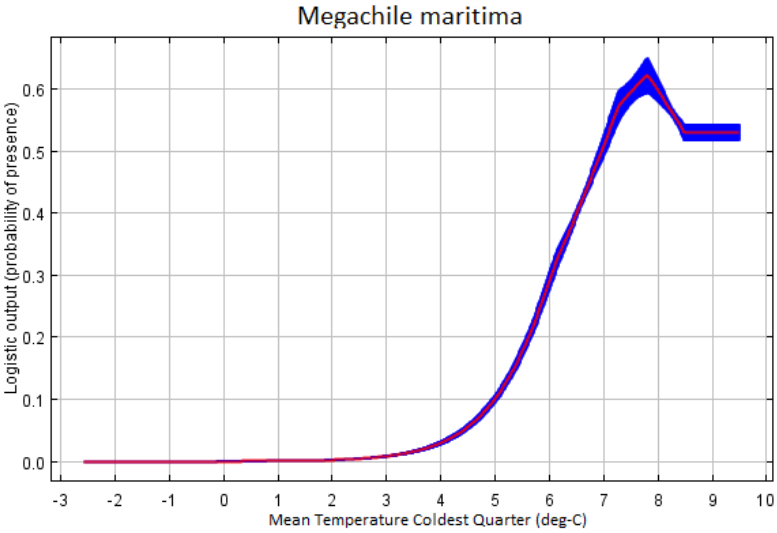

Figure S5-1.4: Predicted probability of occurrence of *Megachile maritima* along the mean temperature of the coldest quarter.

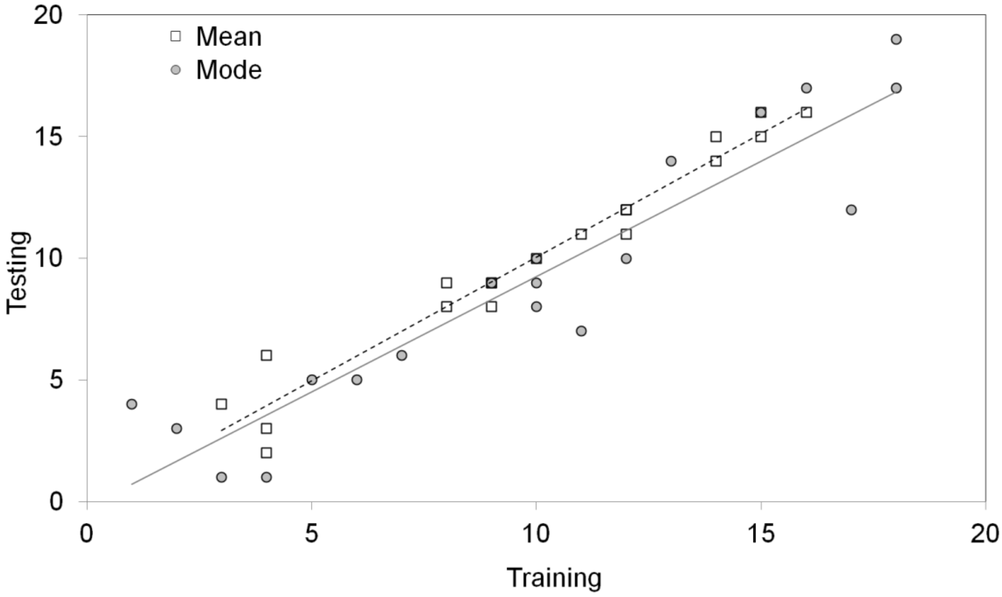

Figure S5-2: Rank correlation within training and testing data, for predictors *Mean* and *Mode*.

Spearman's rank correlations: Mean (open squares and dashed line): ρ = 0.974; Mode (filled circles and solid line): ρ = 0.944. Both correlations are significant, based on 999 bootstrap replicates (Fig. S5-4 panel A and B).

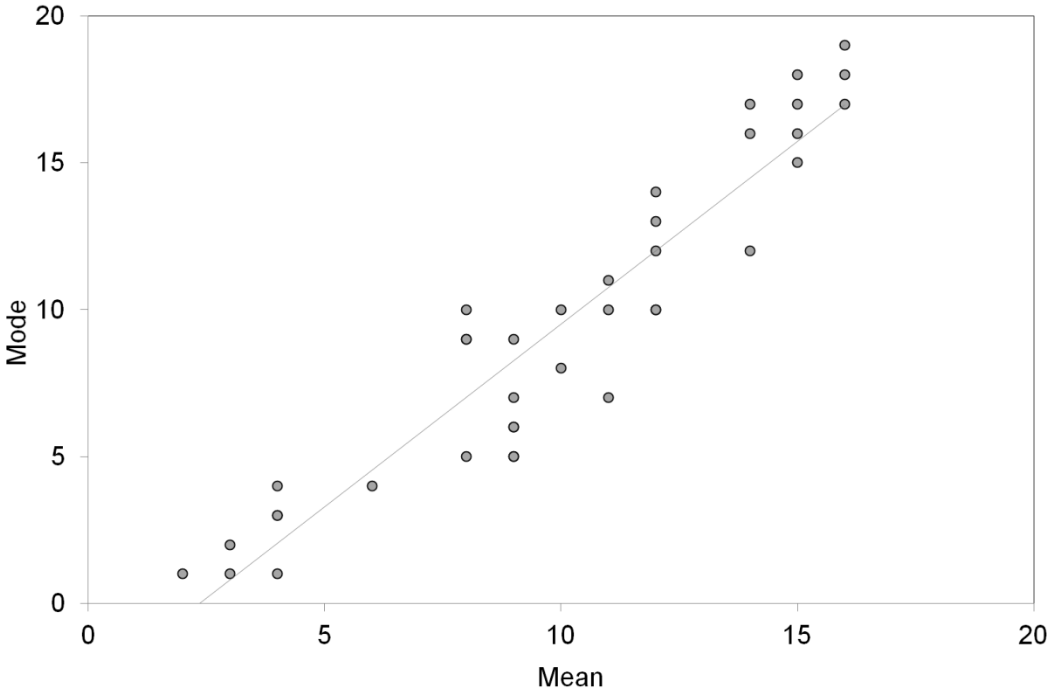

Figure S5-3: Rank correlation between *Mean* and *Mode*, for the pooled set of training and test models across species.

Spearman's rank correlation: ρ = 0.940, significant based on 999 bootstrap replicates (Fig. S5-4 panel C).

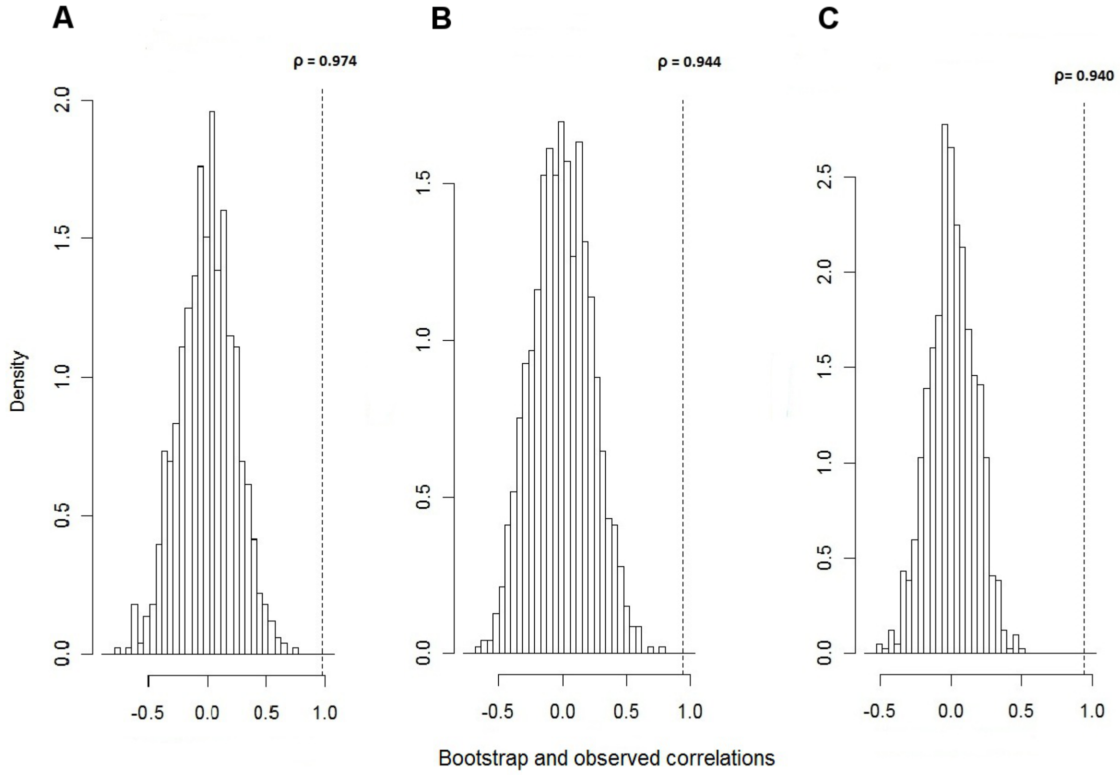

Figure S5-4: Distributions of bootstrap and observed Spearman's rank correlations.

A: correlation of predictors' Mean between training and testing phase; B: correlation of predictors' *Mode* between training and testing phase; C: correlation between *Mean* and *Mode* for the pooled set of training and testing data. In all three cases the observed correlation are significantly greater than those generated from 999 bootstrap replicates.
