# Supplementary material for: Species Distribution Models for Crop Pollination: A Modelling Framework Applied to Great Britain
Source: PLoS One. 2013 Oct 14;8(10):e76308. doi: 10.1371/journal.pone.0076308 (PMC3796555; doi:10.1371/journal.pone.0076308)
Supplement: File S6 — Figure S6–1: Probability of occurrence (A) and potential pollinator service (B) for A. labialis . Figure S6–2: Probability of occurrence (A) and potential pollinator service (B) for A. wilkella . Figure S6–3: Probability of occurrence (A) and potential pollinator service (B) for B. hortorum . Figure S6–4: Probability of occurrence (A) and potential pollinator service (B) for B. lapidarius . Figure S6–5: Probability of occurrence (A) and potential pollinator service (B) for B. lucorum . Figure S6–6: Probability of occurrence (A) and potential pollinator service (B) for B. muscorum . Figure S6–7: Probability of occurrence (A) and potential pollinator service (B) for B. terrestris . Figure S6–8: Probability of occurrence (A) and potential pollinator service (B) for O. rufa . (PDF) [file pone.0076308.s006.pdf]

FILE S6: WILD POLLINATORS' DISTRIBUTION AND POLLINATION SERVICE

Figures from S6-1 to S6-8 show the predicted probability of occurrence and potential pollination service for each of the wild pollinators of field beans in UK. All maps are projected to the British National Grid.

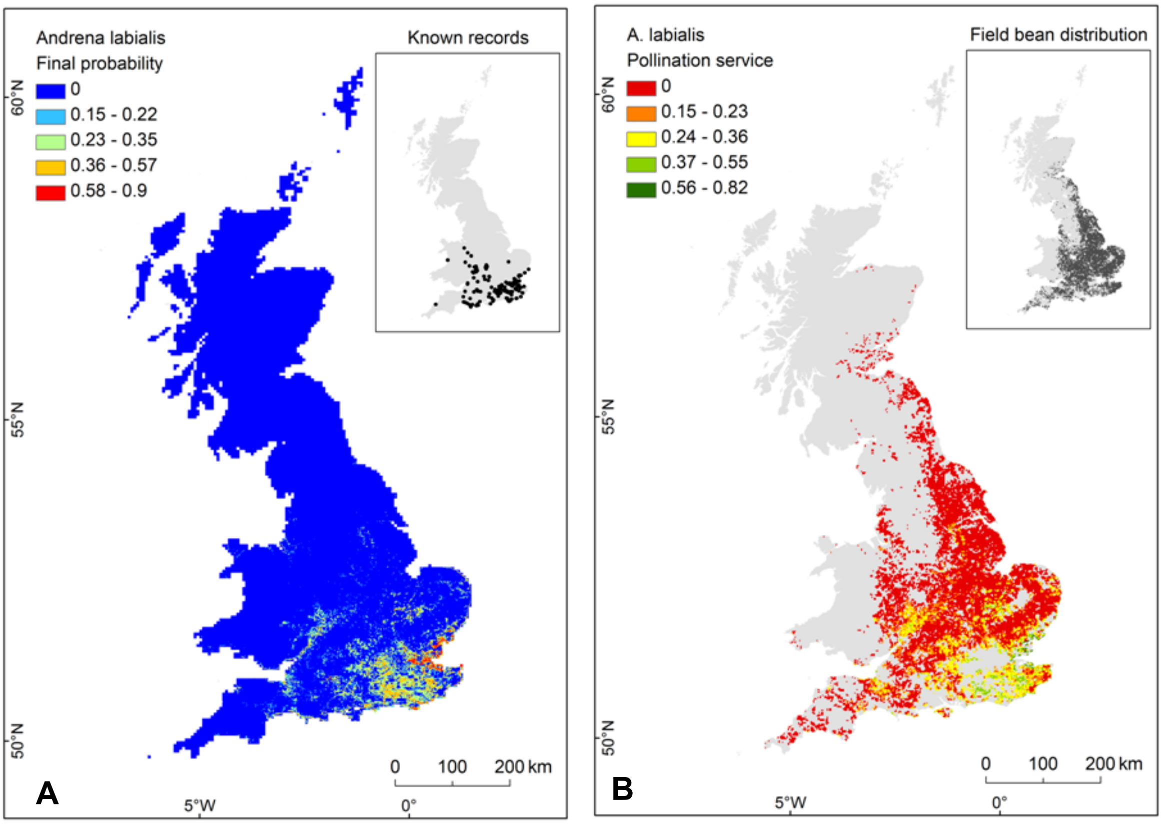

Figure S6-1: Probability of occurrence (A) and potential pollinator service (B) for *A. labialis*.

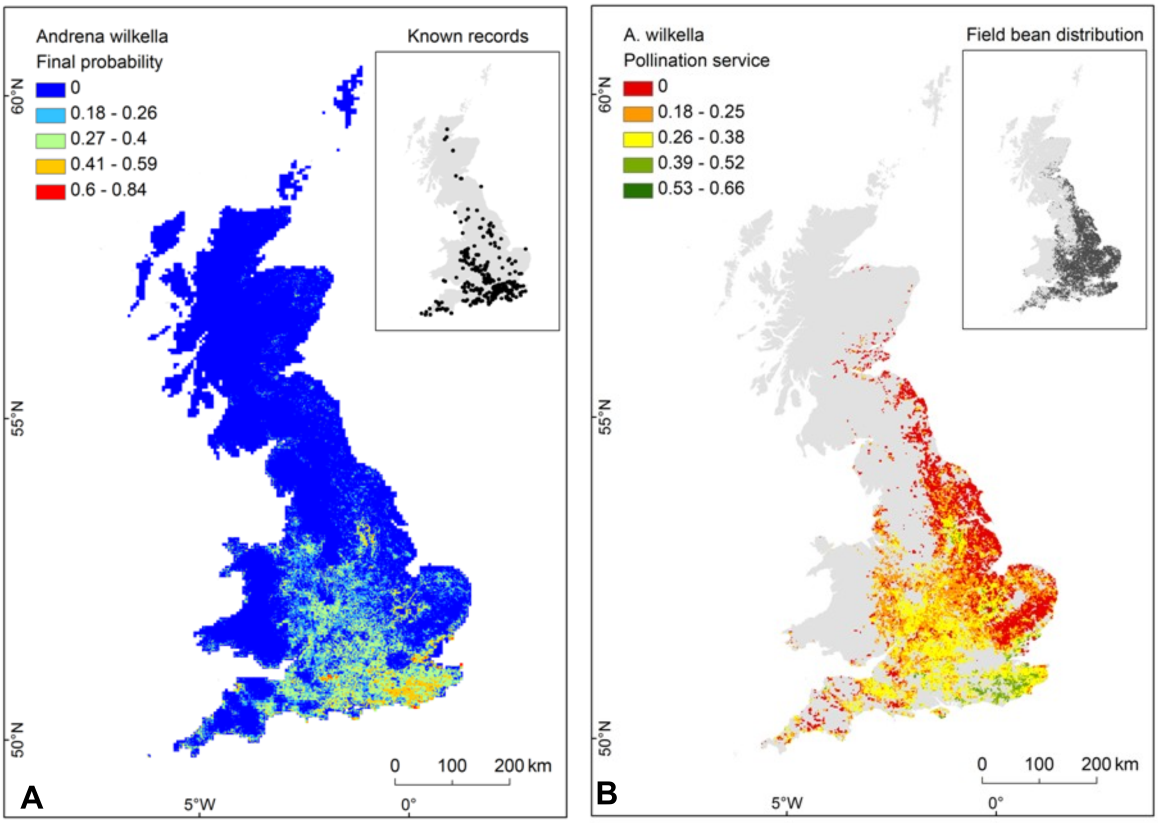

Figure S6-2: Probability of occurrence (A) and potential pollinator service (B) for *A. wilkella*.

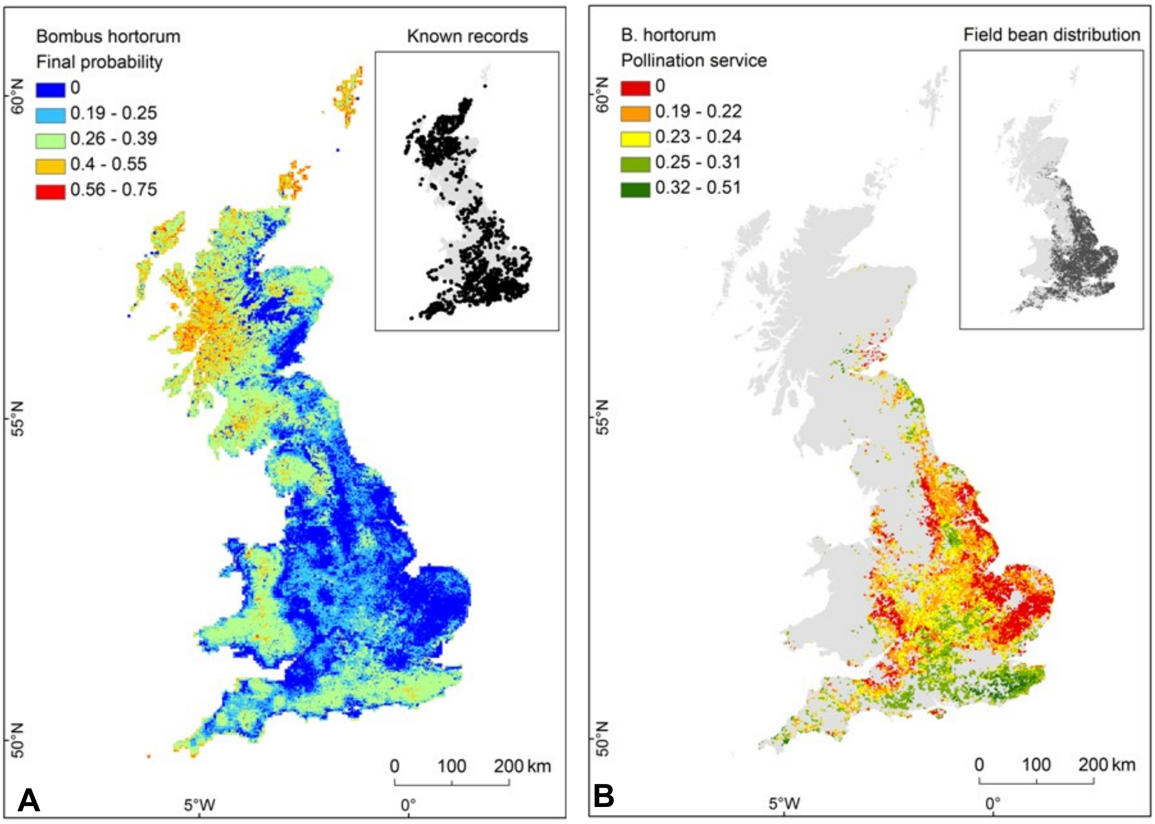

Figure S6-3: Probability of occurrence (A) and potential pollinator service (B) for *B. hortorum*.

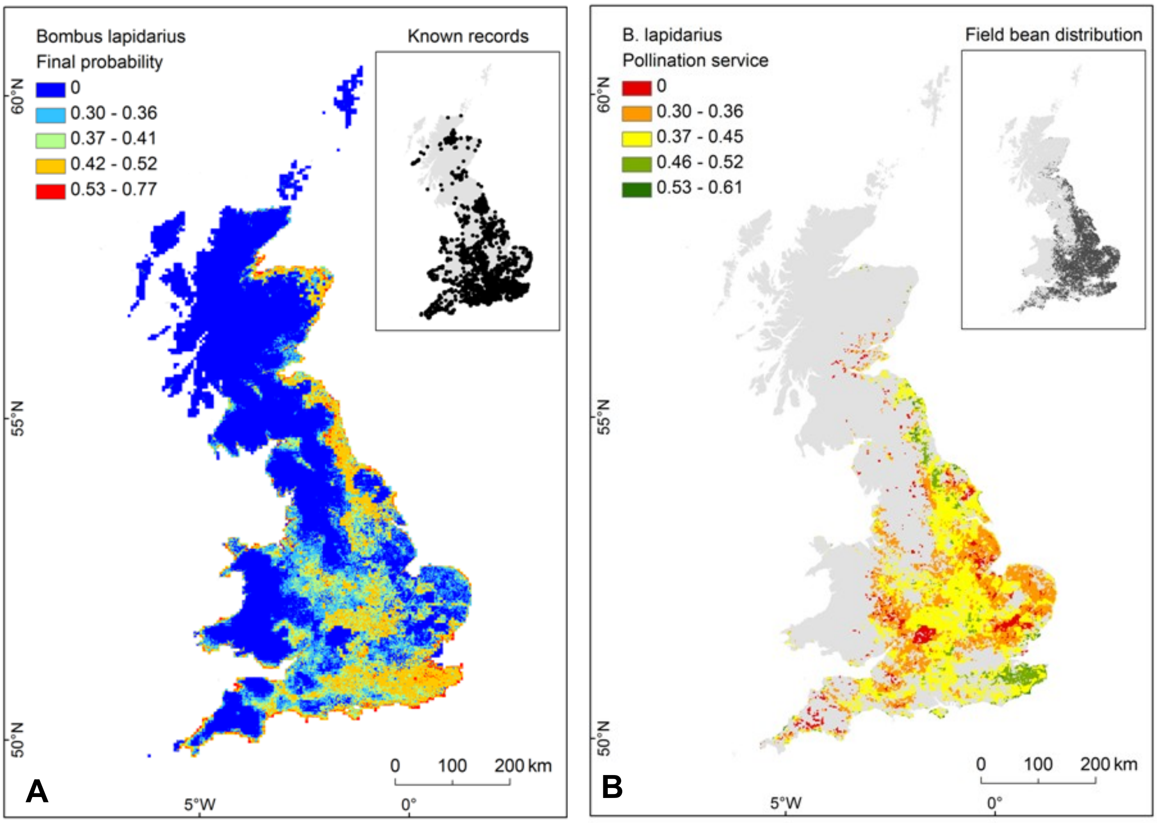

Figure S6-4: Probability of occurrence (A) and potential pollinator service (B) for *B. lapidarius.*

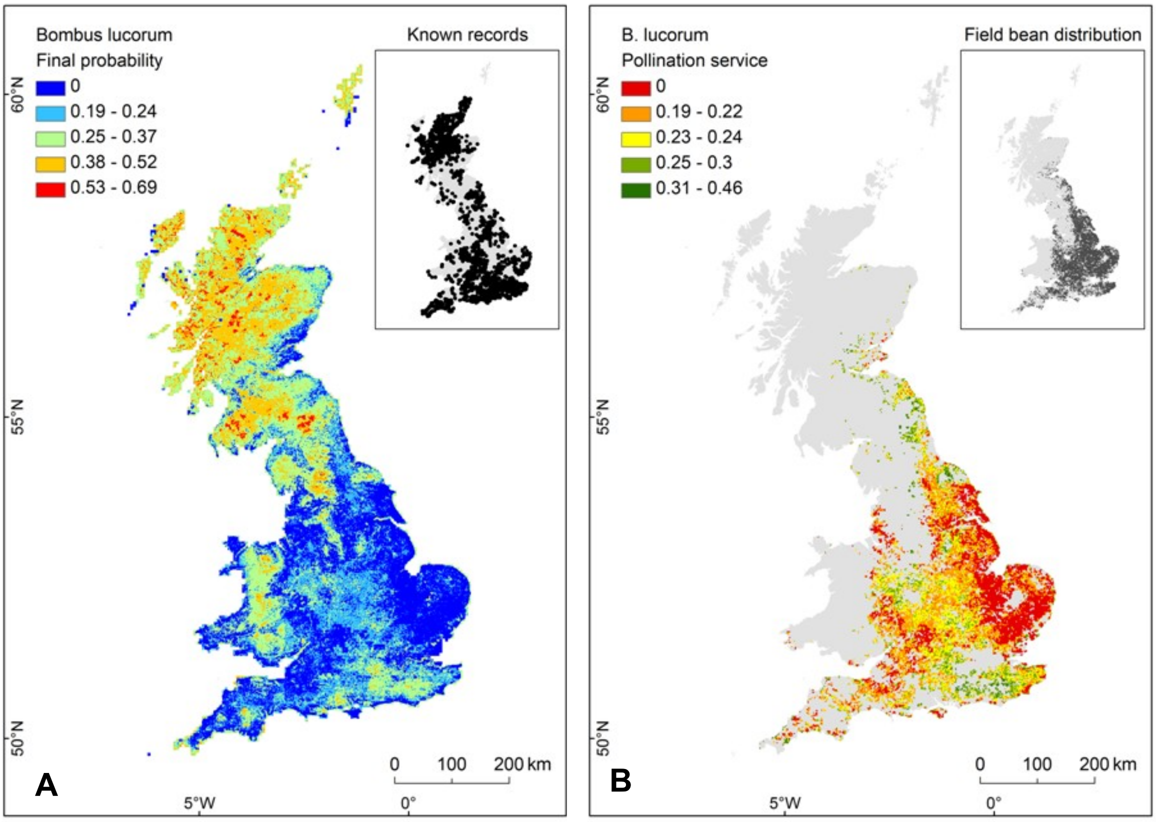

Figure S6- 5: Probability of occurrence (A) and potential pollinator service (B) for *B. lucorum.*

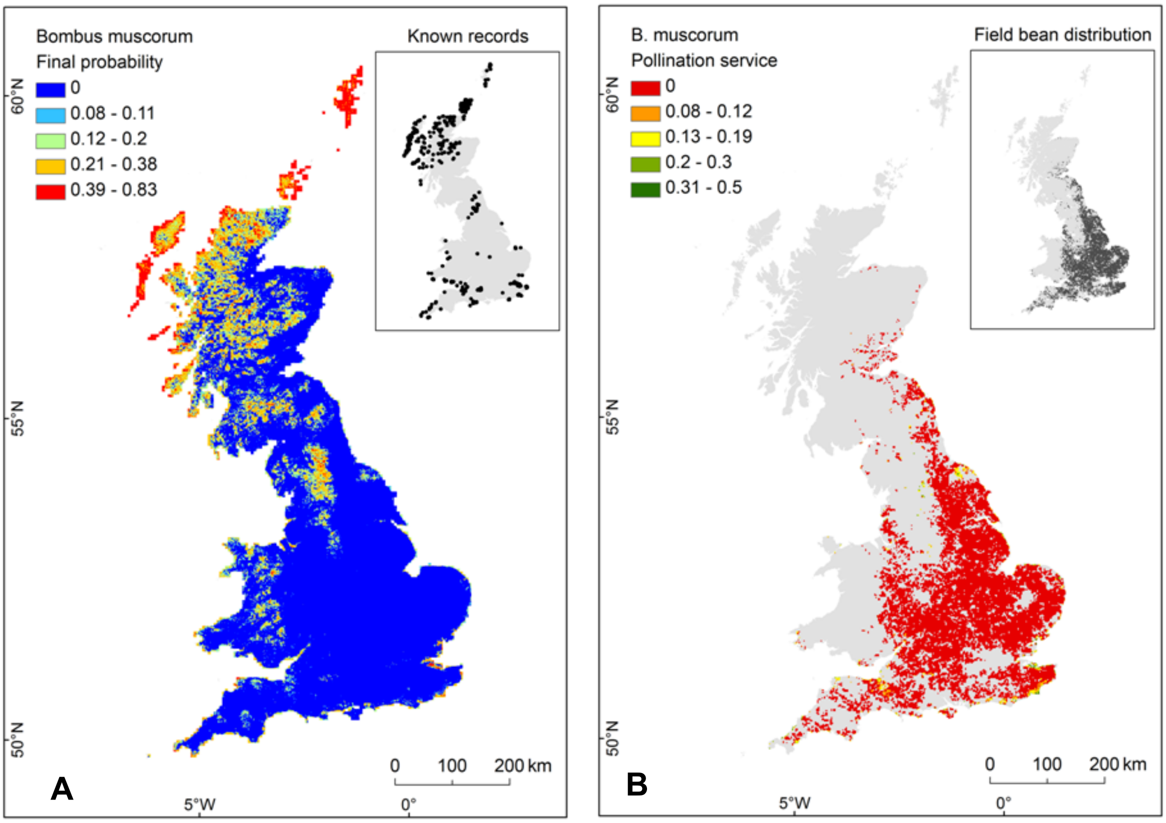

Figure S6-7: Probability of occurrence (A) and potential pollinator service (B) for *B. muscorum*.

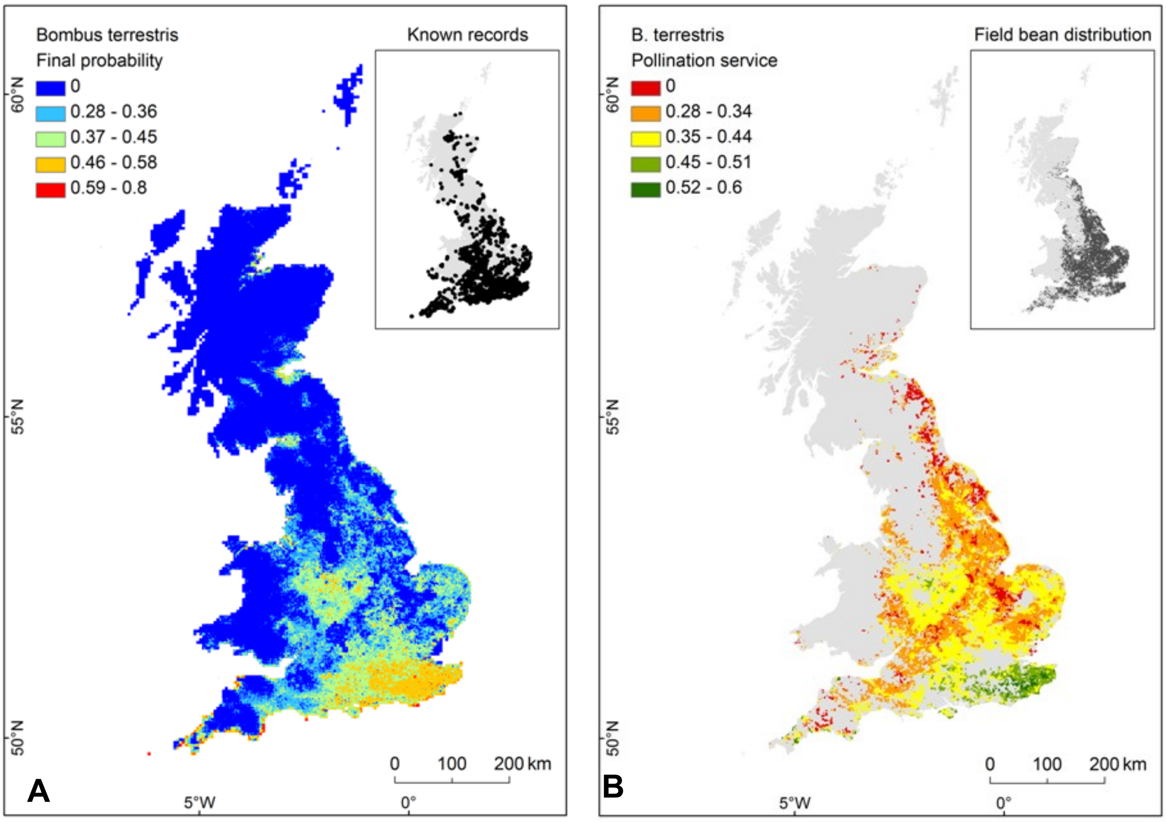

Figure S6-6: Probability of occurrence (A) and potential pollinator service (B) for *B. terrestris*.

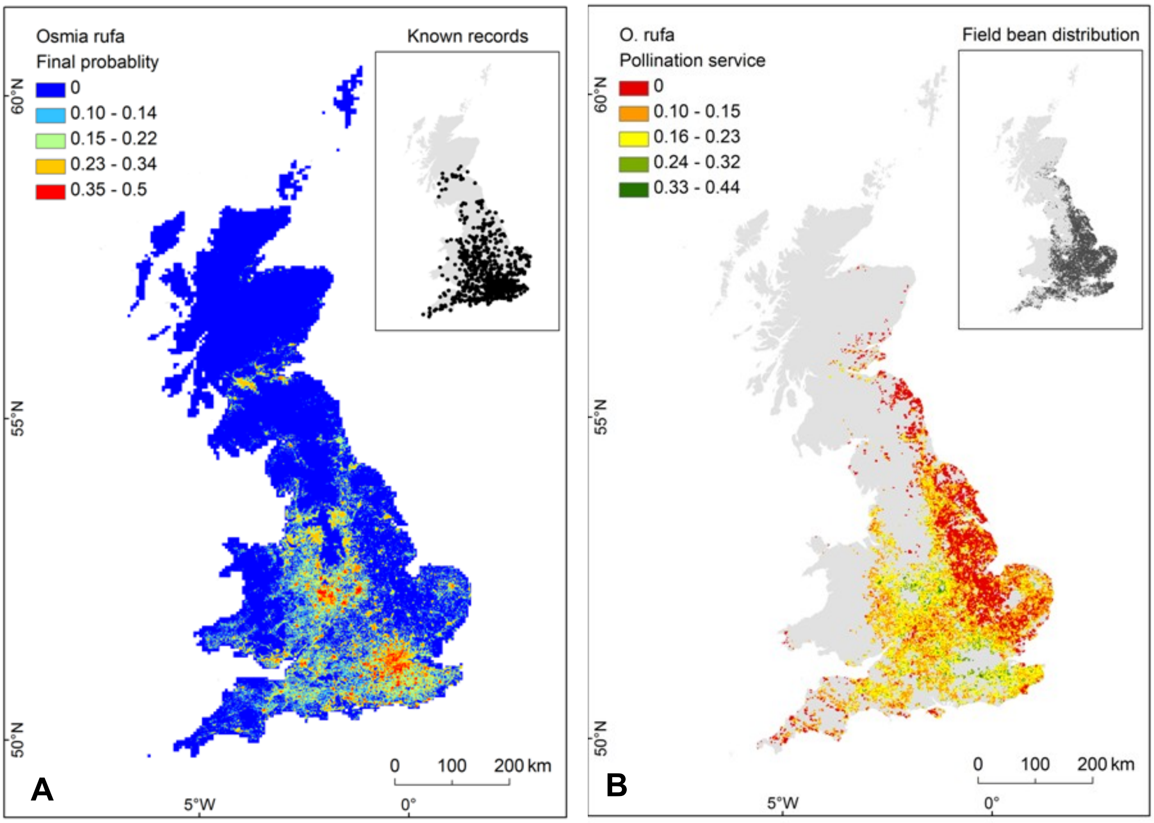

Figure S6-8: Probability of occurrence (A) and potential pollinator service (B) for *O. rufa*.
