# Supplementary material for: Species Distribution Models for Crop Pollination: A Modelling Framework Applied to Great Britain
Source: PLoS One. 2013 Oct 14;8(10):e76308. doi: 10.1371/journal.pone.0076308 (PMC3796555; doi:10.1371/journal.pone.0076308)
Supplement: File S7 — Figure S7–1: Probability of occurrence of managed honey bees. The original density of foragers was linearly rescaled to 0–1 and the 0–1 and the 5th percentile threshold was adopted to distinguish absence from presence (corresponding to a 0.001 probability of occurrence). Map projection: British National Grid. (PDF) [file pone.0076308.s007.pdf]

FILE S7: MANAGED HONEY BEES' DISTRIBUTION

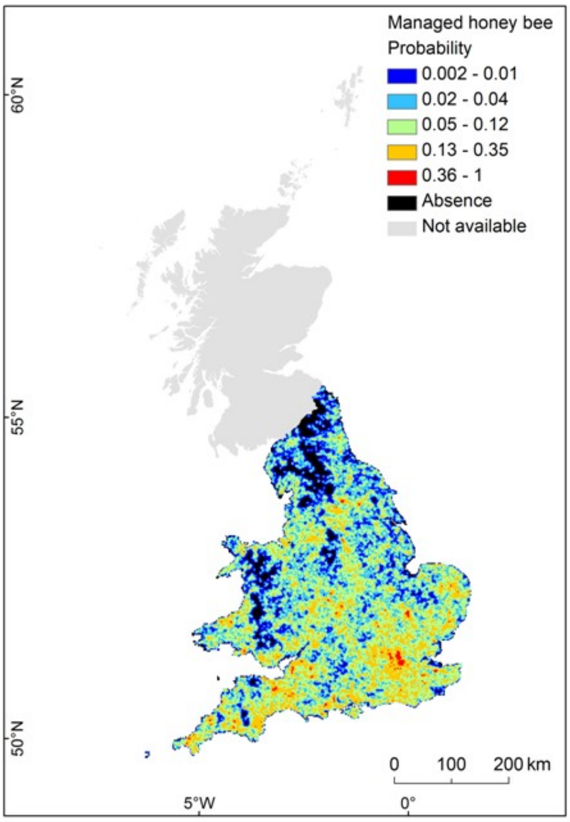

Figure S7-1: Probability of occurrence of managed honey bees.

The original density of foragers was linearly rescaled to 0-1 and the 5th percentile threshold was adopted to distinguish absence from presence (corresponding to a 0.001 probability of occurrence). Map projection: British National Grid.
